# ReDisX, a machine learning approach, rationalizes rheumatoid arthritis and coronary artery disease patients uniquely upon identifying subpopulation differentiation markers from their genomic data

**DOI:** 10.3389/fmed.2022.931860

**Published:** 2022-08-22

**Authors:** Hiu F. Yip, Debajyoti Chowdhury, Kexin Wang, Yujie Liu, Yao Gao, Liang Lan, Chaochao Zheng, Daogang Guan, Kei F. Lam, Hailong Zhu, Xuecheng Tai, Aiping Lu

**Affiliations:** ^1^Computational Medicine Laboratory, Hong Kong Baptist University, Hong Kong, Hong Kong SAR, China; ^2^Institute of Integrated Bioinformedicine and Translational Science, School of Chinese Medicine, Hong Kong Baptist University, Hong Kong, Hong Kong SAR, China; ^3^Department of Mathematics, Hong Kong Baptist University, Hong Kong, Hong Kong SAR, China; ^4^National Key Clinical Specialty, Engineering Technology Research Center of Education Ministry of China, Guangzhou, China; ^5^Guangdong Provincial Key Laboratory on Brain Function Repair and Regeneration, Neurosurgery Institute, Department of Neurosurgery, Zhujiang Hospital, Southern Medical University, Guangzhou, China; ^6^Department of Biochemistry and Molecular Biology, School of Basic Medical Sciences, Southern Medical University, Guangzhou, China; ^7^Department of Psychiatry, First Hospital, First Clinical Medical College of Shanxi Medical University, Taiyuan, China; ^8^Department of Communication Studies, School of Communication, Hong Kong Baptist University, Hong Kong, Hong Kong SAR, China; ^9^Guangdong Provincial Key Laboratory of Single Cell Technology and Application, Guangzhou, China

**Keywords:** precision medicine, redefined diagnosis, genomic signature, ReDisX, machine learning, drug repurposing, continuous max flow

## Abstract

Diseases originate at the molecular-genetic layer, manifest through altered biochemical homeostasis, and develop symptoms later. Hence, symptomatic diagnosis is inadequate to explain the underlying molecular-genetic abnormality and individual genomic disparities. The current trends include molecular-genetic information relying on algorithms to recognize the disease subtypes through gene expressions. Despite their disposition toward disease-specific heterogeneity and cross-disease homogeneity, a gap still exists in describing the extent of homogeneity within the heterogeneous subpopulation of different diseases. They are limited to obtaining the holistic sense of the whole genome-based diagnosis resulting in inaccurate diagnosis and subsequent management. Addressing those ambiguities, our proposed framework, ReDisX, introduces a unique classification system for the patients based on their genomic signatures. In this study, it is a scalable machine learning algorithm deployed to re-categorize the patients with rheumatoid arthritis and coronary artery disease. It reveals heterogeneous subpopulations within a disease and homogenous subpopulations across different diseases. Besides, it identifies *granzyme B* (*GZMB*) as a subpopulation-differentiation marker that plausibly serves as a prominent indicator for *GZMB*-targeted drug repurposing. The ReDisX framework offers a novel strategy to redefine disease diagnosis through characterizing personalized genomic signatures. It may rejuvenate the landscape of precision and personalized diagnosis and a clue to drug repurposing.

## Introduction

Transforming the conventional diagnosis strategy for diseases is emerging to ensure a healthier lifespan (1) and strengthen drug repurposing (2). In the modern era, the practices for diagnosing diseases have been progressively oriented toward precision and personalized, relying on a molecular genetic basis, especially gene expression-based (3, 4). Since the conventional diagnosis of diseases often remains insufficient in explaining heterogeneity within a disease and the homogeneity between multiple diseases (5, 6). Transcriptomic studies demonstrated that the molecular heterogeneity within one disease would be highly divergent, for example, colon cancer (7), breast cancer (8, 9), rheumatoid arthritis (RA) (5), and coronary artery disease (CAD) (6). Alongside the heterogeneity within a disease, the homogeneity among different diseases is also critical to study. For example, RA and CAD have shared a similar inflammatory pathway (10–12). Niu et al. discovered four shared canonical pathways, three shared networks, and three upstream regulators-driven inflammatory activations across RA and CAD (13). Offering symptomatic management without considering the more profound knowledge of underpinning heterogeneity may result in treatment failure and/or resistance to the drugs (14–15).

It certainly embarks us to investigate the initialization of the diseases. Any clinicopathological condition or disease typically originated at the gene level, and the associated altered physiological states and biochemical balances are manifested as phenotypes and symptoms (16, 17). Hence, the clinical symptoms reflect the imbalance of physiological homeostasis. It usually does not comprise the actual underlying disparities at the point of origin, which is at the molecular-genetic layer (16, 17). Therefore, defining the diseases based on clinical symptoms may not encompass the whole underlying disparities at the molecular-genetic level. It is even more unclear when the same gene critically plays a role in manifesting two different disease phenotypes (13, 18, 19), which may lead to an erroneous diagnosis and result in decisive treatments (1). It also hinders the possibility of repurposing some drugs (2). For instance, Aspirin is typically used for managing analgesia, and it was successfully repurposed to manage colorectal cancer as both share the same genetic causes (2).

Enabling precision diagnosis and personalized medicine using unprecedented molecular-level data plays a significant role in modern medicine. The deep multi-view learning approach demonstrated the power of integrated multi-omics data to identify potential biomarkers for specific disease types (20). Several other studies also strengthened the engagement of multi-omics data-driven classifications of the in-disease expression differentiation that elucidates underlying pathological diversities (21, 22). Despite having many advanced tools, most of the available frameworks are merely not adequate to discover the disease heterogeneity. They mostly rely on clustering algorithms that group patients by unsupervised learning upon discovering similar gene expression profiles and then annotating their pathological properties (3, 4). Clustering approaches of both types, deep learning (DL) and machine learning (22) based algorithms have demonstrated their respective competence in investigating the heterogeneity within disease and homogeneity between multiple diseases. However, many existing ML approaches for clustering were slightly out-of-context here as they only focus on heterogeneity within one disease and do not encompass cross-disease similarity within the heterogonous subpopulation. They also fail to specify the optimal number of clusters to be distinctly discovered.

Here, we have proposed ReDisX (Redefining the Disease X) framework to address all those ambiguities described above. It is an integrated ML clustering algorithm relying on a continuous max flow (CMF) model (23, 24). CMF enables the clustering of high-dimensional data with a few training samples without compromising the accuracy (24). It is also less computationally expensive. However, the probability estimation with CMF is not so adaptive, which may affect accuracy sometimes. But, updating the probabilities adaptively in the numerical algorithms enhances accuracy (24). CMF uses a hierarchical clustering approach to pre-label the input patient data and then predicts the patient labels uniquely. ReDisX re-categorizes the diseases based on the heterogeneity of individual gene expression profiles of the patients. Then, it evaluates the cross-disease similarity in expression level to discover whether two different diseases share the same or similar gene expression profiles. This paper considers CAD and RA as our diseases of interest based on prior research experience (13). The ReDisX is self-sufficient in evaluating the optimal number of clusters.

Our results revealed an enhanced differentiation capacity compared to the conventional diagnosis (Figures 2, 4). ReDisX showed distinct differentiation ability across the diversified datasets (Figures 2, 4). It showed that a subpopulation (*n* = 12) of patients with CAD exhibits higher gene expression similarity and functional enrichment similarity to the RA patients (Figure 5). It clustered the similar gene expression profiles of the subpopulation of CAD and RA consecutively to classify the heterogeneity within a disease. Interestingly, a hub-gene was discovered within the CAD and RA subpopulation related to the drug discovery targeting *granzyme B* (*GZMB*) (Figure 5) (25). Our analyses have endorsed *GZMB* as a potential target for developing drugs for those specific subpopulations of CAD and RA.

ReDisX may offer a clue to repurpose any potential drug candidates discovered for CAD that can be validated for RA and vice versa. It reinforces a novel ML approach to redefine the existing two diseases into a total of eight (three within RA and five within CAD) distinct categories based on gene expression signatures. This data-driven novel approach offers an enhanced resolution in introducing better precision and a personalized diagnosis strategy.

## Materials and methods

### Dataset and preprocessing

#### Gene expression data

Two publicly available datasets, GSE93272 and GSE59867, were collected from the National Center for Biotechnology Information Gene Expression Omnibus (NCBI–GEO). The GSE93272 is gene expression data obtained from a whole blood transcriptome (26). It contains 275 samples from RA patients and healthy controls, performed with Affymetrix Human Genome U133 Plus 2 (GPL570). The whole blood transcriptome dataset was retrieved from https://www.ncbi.nlm.nih.gov/geo/query/acc.cgi?acc=GSE93272. The RA patient’s data within this dataset was categorized into three groups: (1) the RA patients without any receiving treatments [pure RA (pRA)], (2) the RA patients receiving treatments such as methotrexate (MTX, responsive as MTX and irresponsive as MTX.IR), infliximab (IFX, responsive as IFX and irresponsive as IFX.IR), and tocilizumab (TCZ, responsive as TCZ and irresponsive as TCZ.IR), and (3) the healthy controls (HCs) (26). The dataset contains a total number of 20,356 genes’ expression values for each patient, which was further processed and normalized using Robust Multi-Array Average (RMA). In our analysis, we labeled all the RA patients with/without drug treatments as RA. We only used the term pRA while demonstrating the performance of ReDisX with/without the influence of drug treatment, as mentioned in one of the test RA datasets, GSE93272. The representation of the dataset is shown in Table 1.

**TABLE 1:** Representation of the datasets used in our study.

	Datasets	Species	Disease	Sample type	Size	Inclusion	Exclusion	Remarks	PMID
Test	GSE93272	Human	RA	Whole blood	275	RA (with/without treatment) HCs	Treatment effects	ReDisX does not get influenced by the treatment (see the Section “Discussion” for details)	30013029
	GSE59867	Human	CAD	Peripheral blood	436	111 STEMI patients: with reported CAD 46 HCs: with a stable CAD and without a history of MI	Four stages of STEMI follow up and age and gender effect	ReDisX does not influenced by the stages or age or gender (see the Section “Discussion” for details)	25984239
Validation	GSE15573	Human	RA	Peripheral blood	33	18 RA 15 HCs	NA	NA	19710928
	GSE77298	Human	RA	Synovial tissue	23	16 RA 7 HCs	NA	NA	26711533
	GSE23561	Human	RA, CAD	Peripheral blood	35	6 RA 6 CAD 9 HCs	6 MS 8 T2D	MS and T2D out of our study interests	21368773

RA, rheumatoid arthritis; CAD, coronary artery diseases; HCs, healthy controls; STEMI, ST-elevation in myocardial infarction; MS, metabolic syndrome; T2D, type-2 diabetes mellitus; PMID, PubMed identifier; NA, not applicable.

The GSE59867 is also a whole blood transcriptome dataset (27). It contains 436 samples from the CAD patients and healthy controls, performed with Affymetrix Human Gene 1.0 ST Array (GPL6244). The data was retrieved from NCBI GEO https://www.ncbi.nlm.nih.gov/geo/query/acc.cgi?acc=GSE59867. The samples were collected from peripheral blood from patients (*n* = 111) with ST-segment elevation myocardial infarction (STEMI). The control group consists of 46 individuals either with reported stable CAD or without any history of MI. The dataset contains a total number of 20,511 gene expression values for each patient, which were further processed and normalized using RMA. The representation of the dataset is shown in Table 1.

#### Data preprocessing

Intersections of the genes (*m* = 17,432) were extracted from both the datasets, GSE93272 and GSE59867. Top 5,000 High Variance Genes (HVGs) were filtered out for further analysis, and the voom (28) was applied separately to estimate the mean-variance relationship between those two datasets.

### Construction of the ReDisX framework and applications

**FIGURE 1:**
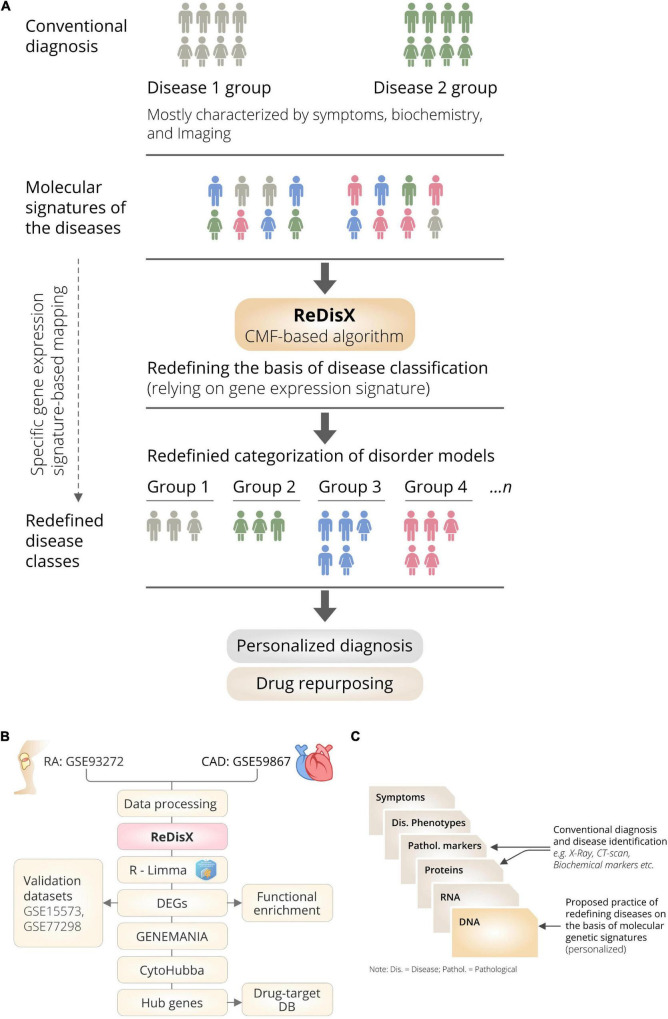
The schematic illustration of the ReDisX framework and its functional features. **(A)** ReDisX-based approach to redefine the disease diagnosis compared to the conventional diagnosis, **(B)** functional pipeline for the ReDisX-based analyses for RA and CAD datasets, **(C)** strategic significance of ReDisX framework over the conventional diagnosis procedure.

ReDisX is an ML framework primarily relying on a CMF model (23, 24). The construction of the ReDisX framework consists of two main parts, (1) hierarchical clustering for pre-labeling all the sample points, and (2) part of the pre-labeling samples would be used in CMF for final labeling for each sample (please see the Section “Weight function” for details) (Figure 1B).

#### Clustering the patients using ReDisX

After sorting out the HVGs, the ReDisX was applied to cluster the patients based on gene expression similarity patterns (see Sections “Notations” for notation, “Hierarchical clustering” for hierarchical clustering, and “The continuous max flow model” for the detailed construction of the CMF model) (Figure 1B). Then, each patient was labeled where the same label represents a higher similarity discovered in their expression profile.

#### Notations

We used lower-case letters such as *x* to represent scalar or single random variables, bold lower-case letters such as ***x*** to represent a vector variable, bold capital letters such as ***X*,** and letters in calligraphy such as 𝒳 to denote a matrix variable and a set, respectively. For example, in this paper, the data set was denoted as a calligraphic capital letter 𝒳={**x**_1_,**x**_2_,…,**x**_*n*_} and **X** = (**x**_1_,**x**_2_,…,**x**_*n*_) ∈ ℛ^*d* × *n*^ is a matrix of the aligned vectors, respectively, while **x_i_** are *d*-dimensional variables, and *n* is the number of the data samples, **x**_*i*_ It is a data point of the *i*-th sample, denoted as a vector. We also used ||⋅|| to measure the length of the vector, and it used Euclidean or *L*_2_ norm by default in the following part if there is no other explanation. In this paper, we used *n* and *K* as the number of samples in the dataset and the number of clusters to be divided, respectively. The notation 𝒞 was used to denote the dataset and 𝒞_*k*_ represents the *k-th* cluster. Normalization of the matrix **X** concerning dimension was a prerequisite to executing the normalization function in MATLAB.

#### Hierarchical clustering

Agglomerative hierarchical clustering is one of the most popular clustering methods. The detail was investigated by (29). It aims to construct a hierarchical partition of data samples using a greedy strategy. In the initial stage, all data samples were viewed as individual clusters. Each turn takes a pair of two nearest clusters and merges them into a larger cluster. Thus, the number of clusters decreased by one after each turn. MATLAB has also implemented agglomerative hierarchical clustering in some **clusterdata** and **linkage** functions. In our experiments, we used Ward’s method, which is also known as the average linkage or minimum variance method, to measure the distance between two clusters, which is defined as follows,


(1) $$dis\left({𝒞}_{i},{𝒞}_{j}\right)=\frac{1}{\left|{𝒞}_{i}\right|\cdot \left|{𝒞}_{j}\right|}\sum_{x_{i}\in {𝒞}_{i}}\sum_{x_{j}\in {𝒞}_{j}}||{\text{x}}_{i}-{\text{x}}_{j}||,$$

where |𝒞_*i*_| represents the number of samples in the *i*-th cluster.

#### The continuous max flow model

The graph models for clustering view the data points as individual vertex and use edge sets to describe the relation between different data points. This well-known model, CMF, was constructed using the max-flow/min-cut theorem (24). It is used to depart the graph into *K-*connected components with minimal cutting edges. The continuous version of the max-flow/min-cut model was termed CMF, which offered an efficient numerical method (24). It minimized the cost of edge cutting and introduced a regional force that could extract the preliminary information from the dataset with other techniques. We used the same specification as stated by (24) in this study, and the region force term was defined in the followings,


(2) $$RF=\sum_{k=1}^{K}\sum_{i=1}^{n}\phi_{k}\left({\text{x}}_{i}\right)f_{k}\left({\text{x}}_{i}\right),$$

where $\phi_{k}(x_{i})=\{\begin{array}{c} 1\;\mathrm{if}\;x_{i}\in {𝒞}_{k},\\ 0\;\mathrm{otherwise} \end{array}$, {*f*_*k*_} is the regional forces. We set that *f*_*k*_(**x**_*i*_) = −*log*(*p*_*k*_(**x**_*i*_))+*log*(1−*p*_*k*_(**x**_*i*_)) likes in (24), where the function {*p*_*k*_} a set of prior conditional probabilities which has been discussed in the Section “Weight function.” The min-cut problem with region force was to minimize the following energy,


(3) $$C=\sum_{k=1}^{K}{||\nabla \phi_{k}\left({\text{x}}_{i}\right)||}_{1}+\sum_{k=1}^{K}\sum_{n=1}^{n}\phi_{k}\left({\text{x}}_{i}\right)f_{k}\left({\text{x}}_{i}\right)$$

(4) $$=\sum_{k=1}^{K}\sum_{i=1}^{n}\sum_{j=i}w_{ij}\left|\phi_{k}\left({\text{x}}_{i}\right)-\phi_{k}\left({\text{x}}_{j}\right)\right|+\sum_{k=1}^{K}\sum_{i=1}^{n}\phi_{k}\left({\text{x}}_{i}\right)f_{k}\left({\text{x}}_{i}\right),$$

where $\sum_{k=1}^{K}{||\nabla \phi_{k}\left({\text{x}}_{i}\right)||}_{1}$ was defined as the term edge cutting, and {*w*_*ij*_} are the weights of the edge between the data points **x**_*i*_ and **x**_*j*_ which was discussed more in the following Section “Weight function.”

Let (*ϕ*_1_, *ϕ*_2_, …, *ϕ*_*k*_) ∈ {0, 1}^*n* × *K*^, and **F** = (*f*_1_, *f*_2_,…, *f*_*k*_) ∈ ℛ^*n* × *K*^, **W** = (*w*_*ij*_) ∈ ℛ^*n* × *K*^, *and* **D** = (**d**_**i***i*_) ∈ **R**^**n** × **n**^ would be explicitly defined in the Section “Weight function,” then the above cost function could also be written as the following,


(5) $$\begin{array}{c} \min_{\Phi \in {\left\{0,1\right\}}^{n\times K}}C=\alpha {||L^{\frac{1}{2}}\Phi ||}_{1}+<\Phi ,F>,\\ \;s.t.\Phi 1=1. \end{array}$$

where **L** = **W**−**D**, ||**A**||_1_ = ∑_*i*, *j*_||**A**_*ij*_|| and α is a regularization parameter that balances the region force and min-cut term. However, searching for the optimal solution **Φ** in discrete space {0,1}^*n* × *K*^ was an **NP**-hard problem, then the feasible space of the above problem was relaxed into a continuous one as Eq. (5) and solved the following problem


(6) $$\begin{array}{c} \min_{\Phi \in {\left[0,1\right]}^{nxK}}C=\alpha {||{\text{L}}^{\frac{1}{2}}\Phi ||}_{1}+<\Phi ,F>,\\ \;s.t.\Phi 1=1. \end{array}$$

Then, this optimal problem becomes equivalent to the min-max problem as Eq. (6),


(7) $$arg\underset{\Phi \in △}{min}\underset{\left|\Psi \right|\leq 1}{max}\alpha <\Psi ,{\text{L}}^{\frac{1}{2}}\Phi >+<\Phi ,F>,$$

where △ = {**Φ** ∈ [0, 1]^*n* × *K*^: **Φ1** = **1**}. We also used the primal-dual method with the projection technique to solve this problem. The details of the algorithm can be found in (24).

#### Weight function

We now describe how to evaluate a prior probability that each data point **x**_*i*_ belong to the *k*-th cluster 𝒞_*k*_, denoted as *p*_*k*_(**x**_*i*_) in Section “The continuous max flow model” In the pre-labeling stage, each datapoint *x_i_* were classified into the *k*-th cluster by the Hierarchical method. Among those data points, part of the would be used as a pre-label for the CMF model, which would be assigned *p_k_*(**x**_*i*_) = 1 and *p*_*j* ≠ *k*_(**x**_*i*_) = 0. The pre-label would be equal for each cluster, and the number of pre-labels for each cluster would be the minimal number of elements in all clusters. For other data points, we introduced a metric on the data that measures the rate of connectivity of the points **x**_*i*_ and **x**_*j*_ based on the m-steps diffusion process (30). The diffusion process could be viewed as a Markov chain, and in our experiment, the transition matrix **A** ∈ ℛ^*n* × *n*^, **A**_*ij*_ means the probability of the data point diffuses from **x**_*i*_ to **x**_*j*_. Let $w\left({\text{x}}_{i},{\text{x}}_{j}\right)=\frac{<x_{i},x_{j}>}{||x_{i}||||x_{j}||}$, then we set ${\text{A}}_{ij}=\frac{w\left(x_{i},x_{j}\right)}{\sum_{j^{\prime }}^{n}w\left(x_{i},x_{j^{\prime }}\right)}$. Denote **D** = (*d*_**i***i*_) ∈ ℛ^*n* × *n*^ is a diagonal matrix where $d_{ii}=\sum_{j=1}^{n}w\left({\text{x}}_{i},{\text{x}}_{j}\right)$, and $\hat{\text{A}}={\text{D}}^{\frac{1}{2}}{\text{AD}}^{-\frac{1}{2}}$ is a symmetric matrix. Then the m-step diffusion distance between *x_i_* and *x_j_* is defined as $D_{m}^{2}\left({\text{x}}_{i},{\text{x}}_{j}\right)={\hat{\text{A}}}_{ii}^{m}+{\hat{\text{A}}}_{jj}^{m}-2{\hat{A}}_{ij}^{m}$.

Here ${\hat{\text{A}}}_{ij}^{m}$ could interpret the transition probability through the m-step diffusion (while ${\text{A}}_{ij}^{m}$ is the real probability), then we define the probability of **x**_*i*_ belong to cluster *k* as


(8) $$p_{k}\left({\text{x}}_{i}\right)=\frac{\frac{1}{\left|{𝒮}_{k}\right|}\sum_{x_{j}\in {𝒮}_{k}}r_{ij}}{\sum_{k^{\prime }}^{K}\frac{1}{\left|{𝒮}_{k^{\prime }}\right|}\sum_{x_{j^{\prime }}\in {𝒮}_{k}}r_{ij}},$$

where 𝒮_*k*_ is a set of labeled data samples in the *k*-th group which we discussed in the preprocessing part, |𝒮_*k*_| is the number of samples in the corresponding group, and $r_{ij}=\frac{{\left({\hat{A}}_{ij}^{m}\right)}^{2}}{{\hat{A}}_{ii}^{m}{\hat{A}}_{jj}^{m}}$ represents the similarity between **x**_*i*_ and **x**_*j*_. In an actual implementation, we chose *m* = 2. Similarly, we also chose the cosine distance to measure the distance between **x**_*i*_ and **x**_*j*_, and ${\text{W}}_{ij}=w\left({\text{x}}_{i},{\text{x}}_{j}\right)=\frac{<x_{i},x_{j}>}{||x_{i}||||x_{j}||}$. Considering the computation cost, we only use the edges between other K-nearest data points for each data point. We chose *K* = 10 using the *K*-nearest neighbor algorithm (KNN), where *K* represents the cluster number.

#### Cluster evaluation

As the core CMF model firmly considers the graph connectivity, it tends to merge two similar groups identified by Hierarchical clustering. Based on the merging cluster property, the ReDisX framework was deployed twenty times by iterating the number of clusters from 1 to 20. Further, the unique number of clusters was evaluated upon merging the graph connectivity as indicated by the CMF model. Then, the most occurrence number of clusters was chosen as the optimal number of clusters.

### Screening the differentially expressed genes

The differentially expressed genes (DEGs) were extracted using the Limma package in R (31), considering the *p*-value < 0.05 and log-fold change > 0.05 for both the datasets GSE93272 and GSE59867. A subpopulation within both the datasets represents a set of DEGs about their respective healthy controls. Then, the DEGs were filtered out for both datasets to extract the overlapping genes across the other subpopulations. The sorted overlapping and non-overlapping genes were considered further for our analyses.

### Functional enrichment analysis

The non-overlapping DEGs for both datasets were subjected to functional enrichment analysis. Those genes were further functionally characterized using (32, 33) to discover the maximum enrichment for DisGeNet (34) disease information and to have the most likely pathways using KEGG (35) pathway analysis.

### Knowledge-based differentially expressed genes network

In parallel to the functional enrichment analysis, a knowledge-based network extraction using GeneMANIA (36) was also performed for both datasets (36). So, a knowledge-based network consisting of the DEGs was constructed for each subpopulation within those two datasets.

### Hub genes identification

After constructing and analyzing the knowledge-based networks, the hub genes within each subpopulation were identified. cytoHubba (37), a dedicated plugin under Cytoscape (v3.9.1) (38), was used to discover the hub genes. In each subpopulation, 20 hub genes were identified.

### Drug target screening

Those identified hub genes (from each subpopulation of both datasets) were examined against the drug bank deposited drug targets (39). Upon screening the pool of hub genes to discover potential drug targets, they were subjected to fit the drug-related gene target matching.

### Validation of the hub genes

The ReDisX-based hub genes’ features, especially the co-expression patterns and expression patterns of the DEGs within those hub genes, were validated using two other publicly available datasets, GSE15573 (40) and GSE77298 (41). The GSE15573 is a whole blood transcriptome dataset (40) containing 33 samples from RA patients and healthy controls obtained by Illumina human-6 v2.0 bead chip (GPL6102). The raw data was retrieved from https://www.ncbi.nlm.nih.gov/geo/query/acc.cgi?acc=GSE15573. Then, the GEO2R tool (42) was applied to check the DEGs for validation. Similarly, another dataset, GSE77298, a synovial biopsies-derived transcriptomic dataset, (41) consists of 16 samples from RA patients and healthy controls obtained using Affymetrix Human Genome U133 Plus 2.0 Array (GPL570). Raw data was retrieved from https://www.ncbi.nlm.nih.gov/geo/query/acc.cgi?acc=GSE77298. Then, GEO2R (42) was also used to check the DEGs for validation.

#### The hub genes network analysis for cross-diseases subpopulation

STITCH^1^ (43, 44) database was applied to examine the hub gene network similarity discovered by cytoHubba (37). It also examines the interdependence among the hub genes across the subpopulation of different diseases. The interactions were identified through text mining, experiments, databases, co-expression, neighborhood, gene fusion, co-occurrence, and prediction with a medium confidence level (0.4).

### Dataset visualization

#### Principal component analyses and t-distributed stochastic neighbor embedding

Principal component analyses (PCA) is one of the most popular and efficient linear approaches for dimension reduction (45, 46). PCA’s singular value decomposition technique is an efficient method for extracting data features in low-dimensional linear subspace (45, 46). However, PCA exposed its limitations for high-dimensional data lying on or near a low-dimensional manifold. Hence, a non-linear technique called t-distributed stochastic neighbor embedding (t-SNE) (47) was also applied (47). As Van der Maaten suggested, t-SNE is also limited in high time complexity (47). In our study, the PCA and t-SNE were used in a combinational scheme to visualize the gene expression data from the patient population in a 2D way. MATLAB (v2021b) was used to execute the functions **pca** and **tsne** in this study.

### Drug-associated network analysis

Open Targets platform^2^ (48) was employed for knowledge extraction for its association with the target genes across CAD and RA. Next, we narrowed down our target to the approved drugs only for the DTGs for the rest of the analyses. Then, the network-level association of the ReDisX identified gene and those DTGs were analyzed using GeneMANIA (36). The considered interactions were consolidated pathways, wiki-pathway, reactome, co-expression, physical interaction, drug-interaction, predicted, co-localization, pathway, shared protein domains, and genetic interactions.

## Results

We have developed a novel framework, ReDisX, an integrated ML algorithm relying on the CMF model. It intends to re-categorize the patient populations and their clinical conditions based on specific gene expression signatures (Figure 1A). Our study considered the RA and CAD patient’s data as we had prior expertise in that regime (13).

The ReDisX considers the gene expression data as an input to execute further analyses to identify the core of the core signature genes, which may correspond to the unique features of a clinical condition for an individual (Figure 1B). Our study indicated a distinctive classification among the patients diagnosed with RA and CAD. It also discovers a subpopulation of patients sharing similar characteristics despite being heterogeneous and diagnosed as a separate disease. Ultimately, ReDisX reinforces a fresh data-driven perception to reconsider the basis of diagnosing the clinical conditions, relying on precision focus at the molecular genetic level (Figure 1C). It showed a clue to navigate a druggable target for a disease where a formula originally discovered for other diseases shares a strength to be validated.

### ReDisX redefines rheumatoid arthritis patients through acquiring personalized and precise molecular-genetic information

**FIGURE 2:**
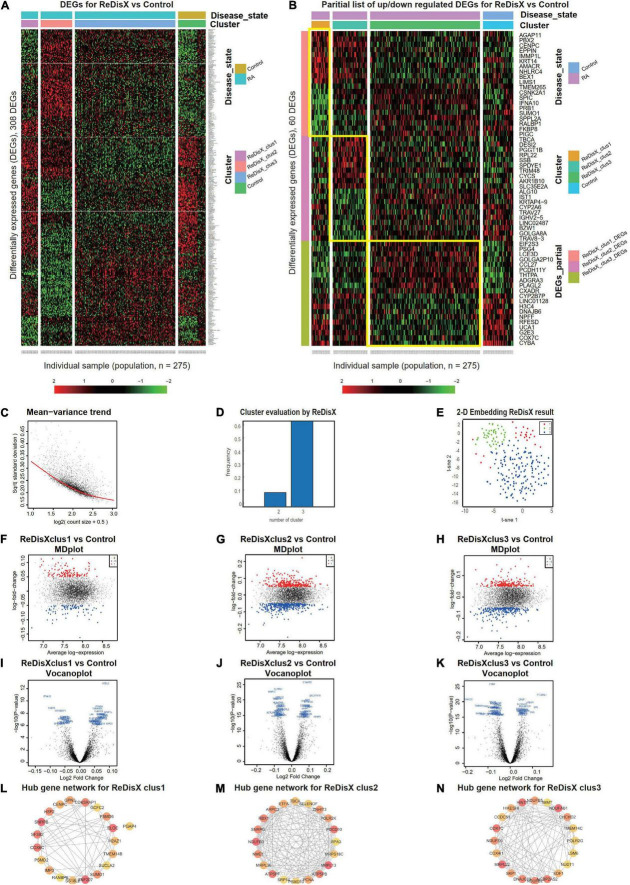
ReDisX redefines the RA patients through acquiring personalized and précised molecular-genetic information. **(A)** Heatmap for relative expression in up/downregulating DEGs identified in GSE93272 (*p*-value < 0.05, log fold change > 0.05, non-overlapping DEGs with other subpopulations). **(B)** Randomly selected 10 up-regulating genes and 10 downregulating genes corresponding to each ReDisX-based cluster in GSE93272 (*p*-value < 0.05, log fold change > 0.05, non-overlapping DEGs with other subpopulation). **(C)** Mean-variance trend after processed voom in GSE93272. **(D)** Optimal subpopulations from the total number of 232 RA patients in GSE93272 by ReDisX. **(E)** PCA and t-SNE visualization of the total number of 232 RA patients in GSE93272. **(F)** Mean-difference (MD) plots for the ReDisX-based cluster 1 of RA patients to the healthy controls [label 1 (red dot) represents the up-regulated genes in the upper panel, the label –1 (blue dot) represents the down-regulated genes at the lower panel]. **(G)** MD plots for ReDisX-based cluster 2 of the RA patients to the healthy controls [label 1 (red dot) represents the up-regulated genes, and the label –1 (blue dot) represents the down-regulated genes]. **(H)** MD plots for ReDisX-based cluster 3 to the healthy controls [label 1 (red dot) represents the up-regulated genes, label –1 (blue dot) represents the down-regulated genes]. **(I)** Volcano plot for ReDisX-based cluster 1 compared to the healthy controls, highlighting top 50 log_2_ fold change genes in blue. **(J)** Volcano plot for ReDisX-based cluster 2 compared to healthy controls, highlighting top 50 log_2_ fold change genes in blue. **(K)** Volcano plot for ReDisX-based cluster 3 compared to healthy controls, highlighting top 50 log_2_ fold change genes in blue. **(L–N)** cytoHubba-identified hub gene analysis to the GeneMANIA network for ReDisX-based clusters using the MCC ranking algorithm. The hub genes for cluster 1 **(L)**, cluster 2 **(M)**, the cluster 3 **(N)** were shown.

ReDisX serves as a core framework to extract the underlying transcriptional heterogeneity of the RA patient population. To identify the optimal number of heterogeneous sub-populations in RA patients, we employed the ReDisX cluster evaluation in human whole blood transcriptome data (GSE93272), which measure the mRNA expression (26). ReDisX adopted the optimal number (*k* = 3) of heterogeneous sub-populations in the RA patients and grouped the high similarity patients into the same cluster (Figure 2D).

As per ReDisX-based patient labeling, differential expressions (DE) analyses were performed on each sub-population using Limma in R (31). After excluding the overlapping DEGs among the subpopulations, a total number of 308 genes were found to be differentially expressed (*p*-value < 0.05, log fold change > 0.05) (Figure 2A). To provide a higher resolution in the co-expression patterns based on the ReDisX discovery, we randomly selected 10 up-regulated genes and 10 downregulated genes for each ReDisX-based cluster. It showed 60 genes in rows and 275 samples in columns (Figure 2B).

Further, the network analyses for those DEGs were conducted using GeneMANIA (36), a Cytoscape function (38). It is constructed using six established co-expression analysis tools (49–53). Next, we estimated the Maximal Clique Centrality (MCC) score that predicts essential nodes within the biological networks in cytoHubba (37). Higher MCC scores indicated the more critical hub genes. Then, the top 20 hub genes were selected from each subpopulation of patients (Figures 2L–N).

The ReDisX framework identified the following hub genes. *SUMO1, PSMD6, SNRPB, H2AZ1, RANBP6, SUCLA2, GPN3, PGAP4, CENPC, PSMD2, ZNF207, GCFC2, SS18L2, COX6C, ELOC, TMEM14B, HSF2, IMP3, CDK5RAP1* for the cluster 1 (Figure 2L), *ATP5PB, MRPS18C, MRPL36, SRP14, PSMD10, NDUFB3, TBCA, ZNHIT3, NME1, RPA3, RBX1, MRPL13, SELENOF, PDCD10, PCNA, ATP5PF, ETFA, SNRPG, ARPC3, POLR2K* for the cluster 2 (Figure 2M), and *COX4I1, SKP1, DNAJC19, NDUFB2, NCBP2AS2, EDF1, IMMT, POLR2G, COX7C, NUDT1, HIKESHI, CCDC51, NDUFB5, MRPL22, HINT1, TMEM14C, LSM6, CHCHD2, NDUFS6, NDUFAB1* for the cluster 3 (Figure 2M) were identified and their corresponding networks were constructed (Figures 2L–N). The detailed Cytoscape networks are available in Supplementary Material 2.

Visualization of the gene expression profiles for RA patients was reduced to 50 dimensions by PCA (45, 46), then further reduced to two dimensions by t-SNE (47). It returned a final two dimensions plot consisting of 232 patients’ data (Figure 2E). Finally, quality control of the data was ensured using a mean-variance trend (Figure 2C). To support it, Mean Difference (MD) plots were plotted for each ReDisX-based cluster to the healthy controls (Figures 2F–H). The volcano plots for each ReDisX-based cluster compared to the healthy controls also highlighted the top 50 log_2_ fold change genes in blue (Figures 2I–K).

### ReDisX output did not interfere with the drug treatments in the rheumatoid arthritis dataset

**FIGURE 3:**
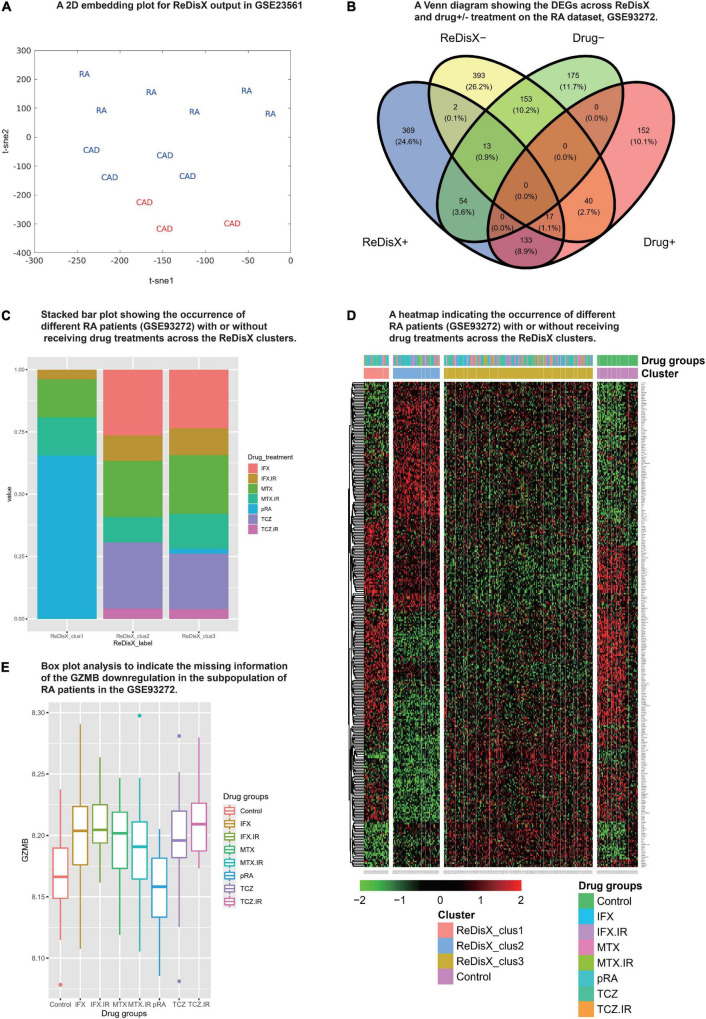
Cross-dataset validation of ReDisX performance to identify the hub genes, and generosity of ReDisX to identify DEGs. This figure comprises two distinct sections, **(A)** indicates the cross-dataset validation of ReDisX performance to identify the hub genes from GSE23561; and **(B–E)** indicates the generalized performance of ReDisX to identify the DEGs on the RA dataset, GSE93272, which includes the RA patients with/without receiving drug treatments. The explanations for the individual legends are following. **(A)** PCA and t-SNE visualization of ReDisX labeling of the total number of 13 patients as reassigned by ReDisX in GSE23561 (for text; RA represents the GSE23561 label as RA patient; CAD represents GSE23561 label as CAD patient) (for color; blue indicate the patient cluster as ReDisX cluster 1; red indicate the patient cluster as ReDisX cluster 2). **(B)** The Venn diagram for the DEGs discovered by the ReDisX label (ReDisX +/–) and drug treatment group label (Drug+/–) (“+” indicate the up-regulated DEGs; “–” indicates the down-regulated DEGs). **(C)** The stacked bar plot that shows the distribution of different drug treatments within the ReDisX labeled clusters (*x*-axis: ReDisX clusters; *y*-axis: percentage of the patients receiving the different drug treatment). The color-coded legends “drug_treatment” represents the different drug treatment groups as recorded in GSE23561, IFX, MTX, and TCZ indicates the responsiveness for the corresponding drugs; IFX.IR, MTX.IR, and TCZ.IR means irresponsive to the corresponding drugs, and pRA means RA patients without treatments. **(D)** The heatmap for relative expressions of the up/downregulating DEGs identified in GSE93272 indicated each patient-specific drug group (*p*-value < 0.05, log fold change > 0.05, non-overlapping DEGs with other subpopulations). **(E)** The box plot shows the *GZMB* expression based on different drug groups (*x*-axis and the legend show the different drug treatment groups as recorded in GSE23561, IFX, MTX, and TCZ indicate the responsiveness for the corresponding drugs; IFX.IR, MTX.IR, and TCZ.IR means irresponsive to the corresponding drugs, and pRA means RA patients without treatments; Control indicates healthy control. The *y*-axis indicates the *GZMB* expression).

We analyzed the propositions of selecting the particular dataset and the rationale for the rest of the analyses used in our study. Our analyses showed that the drug-induced upregulated or downregulated genes (within the RA patients dataset) did not significantly interfere with the ReDisX-discovered DEGs. The deployment of ReDisX merely needs a dataset to be labeled as any disease per conventional diagnosis. Now, to investigate whether the drug treatment affects gene expression, our analyses suggested that the drug treatment certainly interferes with a certain % of gene expressions in the concerned datasets. However, their occurrence within the ReDisX-based discovery of DEGs was <10% (mean interference was 7.35%) in all probable conditions, which is statistically not so significant to be considered in the function of ReDisX deployment on the datasets. Based on our additional analyses, the total estimated interference of ReDisX+ (up-regulated genes detected by ReDisX) and Drug+ (up-regulated genes by drug treatment in GSE93272) subset was 10%, ReDisX+ and Drug− (down-regulated genes by drug treatment in GSE93272) was 4.5%, ReDisX− (down-regulated genes detected by ReDisX) and Drug+ was 3.8%, and ReDisX− and Drug− was 11.1% (Figure 3B). Thus, ReDisX discovered 90% of the upregulated genes irrespective of the drug treatment impacts.

Moreover, to strengthen our claim about ReDisX’s performance, we further analyzed it with a stack bar analysis wherein *X*-axis denotes ReDisX-based clusters and *Y*-axis denotes the percentage of RA patients with/without drug treatment. Our analysis again indicated that the drug treatments (as included in the RA dataset, GSE93272) do not affect the ReDisX results. As indicated in (Figure 3C), each ReDisX cluster contains a mixture of multiple drug treatments. So, the ReDisX-labeled DEGs are not similar to the DEGs with drug treatments. It was further supported with a clustering heatmap (Figure 3D). For example, in the heatmap, the gene at row 1, *ATP6V1F*, is downregulated in all the given drug treatments (IFX.IR, MTX, MTX.IR) and pure RA (pRA). So, it would not be considered a DEG under the influence of the drug treatment. However, *ATP6V1F* is considered a DEG by the ReDisX label. Consequently, the gene *ATP6V1F*, for example, would not be affected by the drug treatment. So, the drug treatment in the RA dataset did not interfere with the ReDisX results as the ReDisX-labeled DEGs were not similar to the DEGs with the drug treatments mentioned in the dataset, GSE93272.

### ReDisX distinguishes the heterogeneous subpopulation among the coronary artery disease patients

**FIGURE 4:**
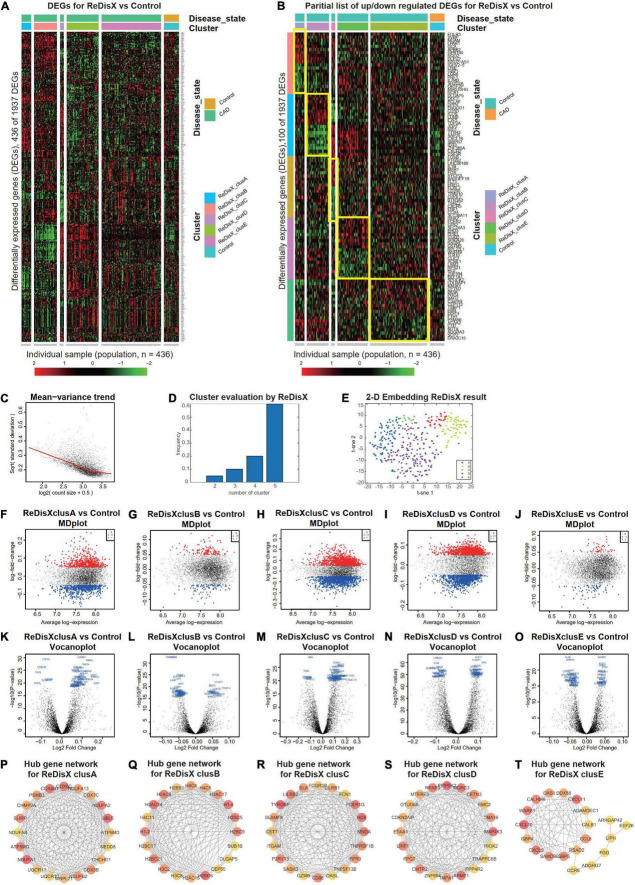
ReDisX distinguishes the heterogeneous subpopulation among CAD patients. **(A)** Heatmap for relative expression in up/downregulating DEGs identified in GSE59867 (*p*-value < 0.005, non-overlapping DEGs with other subpopulations). **(B)** Randomly selected 10 up-regulating genes and 10 downregulating genes correspond to each ReDisX-based cluster in GSE59867 (*p*-value < 0.005, non-overlapping DEGs with other subpopulations). **(C)** Mean-variance trend after processed voom in GSE59867. **(D)** Optimal subpopulations from the total number of 390 CAD patients in GSE59867 by ReDisX. **(E)** PCA and t-SNE visualization of the total number of 390 patients in GSE59867. **(F–J)** MD plots for ReDisX-based clusters. The ReDisX-based cluster A **(F)**, cluster B **(G)**, cluster C **(H)**, cluster D **(I)**, and the cluster E **(J)** of the CAD patients to the healthy controls were shown to have up/down-regulated genes (red dot represent the upregulated genes in the upper panel and the blue dot represents the downregulated genes in the lower panel). **(K–O)** Volcano plots for the ReDisX-based clusters compared to the healthy controls were highlighted using the top 50 log_2_ fold change genes in blue. ReDisX-based cluster A **(K)**, cluster B **(L)**, cluster C **(M)**, cluster D **(N)**, and cluster E **(O)** were shown to have a comparative difference of those top 50 log_2_ fold change genes to the healthy controls. **(P–T)** cytoHubba-identified hub gene analysis to the GeneMANIA network for ReDisX-based clusters using the MCC ranking algorithm. The hub genes for cluster A **(P)**, cluster B **(Q)**, cluster C **(R)**, cluster D **(S)**, and cluster E **(T)** were shown.

Our results also showed the efficiency of ReDisX in extracting the underlying transcriptional heterogeneity of CAD patients. The analysis pipeline was similar to the previous section on analyzing RA patients. To identify the optimal number of heterogenous subpopulations in CAD patients, we employed the ReDisX cluster evaluation in human whole blood transcriptome data (GSE59867) that measures the mRNA expression (27). ReDisX adopted the optimal number (*k* = 5) for heterogeneous subpopulations in the CAD patients and grouped the highly similar patients into the same cluster (Figure 4D).

Then, DE analyses were performed on each subpopulation within the ReDisX-based patient labeling using Limma in R (31). After excluding the overlapping DEGs among the subpopulations, a total number of 436 out of 1937 genes were found to be differentially expressed (*p*-value < 0.005) (Figure 4A). To provide a higher resolution in the co-expression patterns based on the ReDisX discovery, we randomly selected 10 up-regulated genes and 10 downregulated genes for each ReDisX-based cluster. It showed 100 genes in rows and 436 samples in columns (Figure 4B).

Furthermore, the network analyses conducted for other filtered DEGs (*p*-value < 0.05, log fold change > 0.05) were conducted using GeneMANIA (36). We estimated the MCC score to predict the critical nodes within the biological networks (37). Higher MCC scores indicated the more essential hub genes here as well. Then, the top 20 hub genes were selected from each subpopulation of patients (Figures 4P–T) for further analyses.

The ReDisX framework identified the following hub genes. *MRPL21, UQCR10, NDUFB2, COX5B, CHCHD1, NEDD8, COX7C, CHMP2A, NDUFA8, SLIRP, PSMB3, NDUFA1, UQCR11, COX6C, COX6B1, ATP5MG, UBL5, NDUFA2, NDUFA13, ATP5MD* for the cluster A (Figure 4P), *H3C2, H3C8, H2AC16, H2BC6, CEP55, DLGAP5, H2BC9, H2BC5, H1-4, H2BS1, BUB1B, H1-2, H2AC8, H2BC21, H2AC17, H4C5, H4C4, H2AC14, H4C11, H2BC17* for the cluster B (Figure 4Q), and *TGAM, TNFSF13B, CD86, FPR3, P2RY13, GZMB, OASL, TNFRSF1B, SASH3, HCK, FCER1G, FCN1, CST7, LILRB2, LILRB1, TYROBP, MNDA, FCGR3B, SLAMF8, SLA* for the cluster C (Figure 4R), *PPP4R2, TRAPPC6B, RIOK2, ZNF654, IMPA1, RPAP3, EMC2, LRIF1, FPGT, TMA16, MORC3, PPIP5K2, OTUD6B, ARMT1, MAP4K3, ETAA1, CDKN2AIP, MTERF3, CETN3, CMTR2* for cluster D (Figure 4S), *LIPH, ADGRG7, CXCL10, CXCL9, SAMD9L, CALHM6, ARHGAP42, DDX58, GBP5, EEF2K, WARS1, RSAD2, CCL8, FGG, ADAMDEC1, CXCL11, GBP4, CCR9, CALB1, OAS1* for cluster E (Figure 4T) were identified and their corresponding networks were constructed (Figures 4P–T). The detailed Cytoscape networks are available in Supplementary Material 1.

Visualization of the gene expression profiles for CAD patients was reduced to 50 dimensions by PCA (45, 46), then further reduced to two dimensions by t-SNE (47). It returned a final two dimensions plot consisting of 436 patients’ data (Figure 4E).

Finally, quality control of the data was ensured using a mean-variance trend (Figure 4C). MD plots were plotted for each ReDisX-based cluster against the healthy controls (Figures 4F–J). Moreover, the volcano plots for each ReDisX-based cluster compared to the healthy controls were also shown, highlighting the top 50 log_2_ fold change genes in blue (Figures 4K–O).

### ReDisX discovers the cross-subpopulation homogeneity among the rheumatoid arthritis and coronary artery disease patients embarking on the redefinition of their diagnosis at molecular-level

**FIGURE 5:**
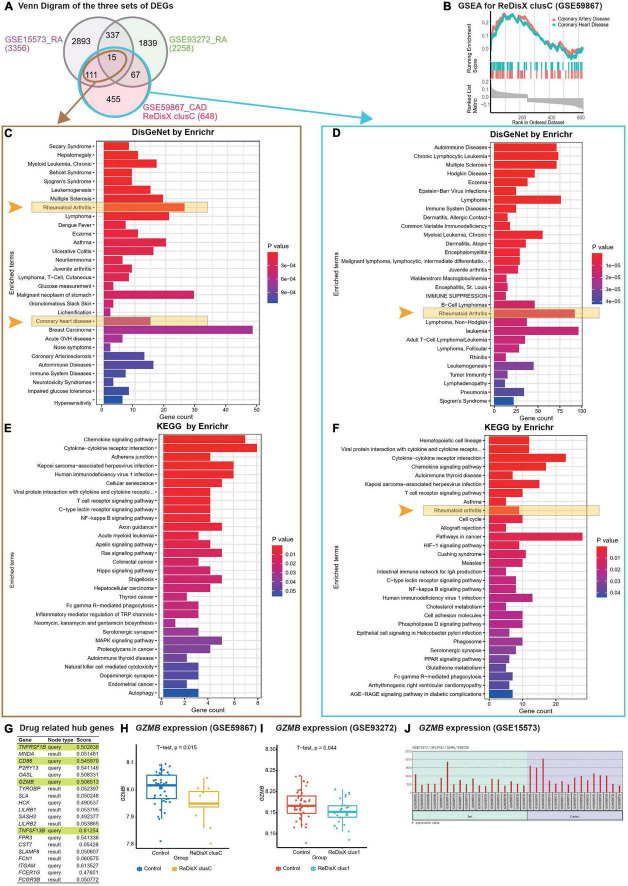
ReDisX-based discovery of the subpopulation homogeneity across the RA and CAD patients. **(A)** Venn diagram of three sets of DEGs, GSE15573 (RA), GSE93272 (RA), GSE59867 (CAD) cluster C. **(B)** GSEA of ReDisX-based cluster C in GSE59867. **(C)** Disease Ontology (DO) enrichment using DisGeNet for the intersecting DEGs of GSE59867 (CAD) cluster C and GSE15573 using Enrichr. **(D)** DO enrichment of GSE59867 (CAD) cluster C DEGs. **(E)** Pathway enrichment for the intersecting DEGs of GSE59867 (CAD) cluster C and GSE15573 by KEGG. **(F)** Pathway enrichment for GSE59867 (CAD) cluster C DEGs by KEGG. **(G)** The list of drug-gene target-related identified hub genes from cluster C of GSE59867 (CAD). **(H)** Boxplot of *GZMB* expression in GSE59867, Control (blue), and ReDisX-based cluster C (yellow). **(I)** Boxplot of *GZMB* expression in GSE93272, Control and ReDisX-based cluster C (blue). **(J)** The expression value of *GZMB* within the patient and healthy controls from GSE15573.

ReDisX revealed the cross-subpopulation homogeneity among the CAD and RA patients by analyzing their transcriptomic profiles (Figure 5). The extent of the homogeneity was assessed in terms of the intersection of DEGs (Figure 5A), functional enrichment analysis (Figures 5B–F) (32, 33), and the drug bank (Figure 5G) (39) across the sub-population of CAD and RA.

Redefining the Disease X-based CAD cluster C (12 patients) of GSE59867 was discovered to be homogeneous to RA patients. Based on ReDisX-based patient labeling, 648 DEGs were identified from the GSE59867 CAD cluster C (Figure 5A). To validate the homogeneity of GSE59867 cluster C to RA, we employed two publicly available validation datasets for RA, GSE15573, and GSE93272, retrieved from whole blood transcriptome. Then, GEO2R (42) was applied to identify the DEGs with default parameters (*p*-value < 0.05). A total of 3356 and 2258 DEGs were identified from GSE15573 and GSE93272, respectively (Figure 5A).

Moreover, a total number of 126 genes were found at the intersection of GSE59867 CAD cluster C and GSE15573 RA. It sparked a notion further to investigate the homogeneity between CAD subpopulation and RA patients, especially to identify some clues about more precision yet personalized diagnosis and drug repurposing. Additional GO analysis is available in (Supplementary Material 2.1). Then, we conducted Gene Set Enrichment Analysis (GSEA) using clusterProfiler (54) to ensure that the ReDisX framework does not lose any essential molecular functions associated with the identified DEGs from the subpopulation of cluster C of CAD patients (Figure 5B).

Further to examine the hypothesis above, a total number of 126 intersecting DEGs of GSE59867 CAD cluster C and GSE15573 RA were analyzed for their functional enrichment using Enrichr (32, 33) and also with two other databases, DisGeNet (34), to identify the enrichment of diseases, and KEGG (35) for common pathways. In the DisGeNet enrichment analysis of 126 intersecting DEGs, RA and coronary heart disease, which implies a broader term for CAD, were observed in the top 30 enriched Disease Ontology (DO) terms. Also, in the KEGG analysis of 126 intersecting DEGs, two essential inflammatory pathways, Chemokine signaling, and Cytokines-Cytokines receptor interaction were observed in the top two enriched KEGG terms. Additional GO analysis is available in (Supplementary Material 2.2). Similar validations were also conducted for another RA dataset, GSE77298, from synovial biopsies tissue (Supplementary Material 3).

In parallel to the intersecting DEGs of GSE59867 CAD cluster C and GSE15573 RA (Figure 5A), the functional enrichment of a total number of 648 DEGs of GSE59867 cluster C was analyzed using Enrichr (32, 33) with two databases, DisGeNet (34) and KEGG (35). In the DisGeNet enrichment analysis of those DEGs, we observed that RA was in the top 30 enriched DO terms. Also, In the KEGG enrichment analysis of those DEGs, we observed that RA, Chemokine signaling, and Cytokines-Cytokines receptor interaction were in the top 10 enriched KEGG terms.

To identify whether any distinguished subpopulation shares drug-related targets and/or pathways, we employed the drug bank (39) to retrieve the drug-gene target database to analyze the drug-related hub genes of cluster C of GSE59867 CAD. Of them, 4 genes (*TNFRSF1B, CD86, GZMB, and TNFSF13B*) were identified as the drug-related hub genes (Figure 5G, highlighted in light green). Our analysis indicated that one of the drug-related hub genes, *GZMB* was under-expressed in the ReDisX-based cluster 1 of RA patients and the ReDisX-based cluster C of CAD patients. The gene *GZMB* encodes the granzyme B, secreted by natural killer cells and cytotoxic T-lymphocytes to induce inflammatory reactions by processing cytokines and imparting into chronic inflammations, including RA (55) and cardiovascular diseases (25, 56). It plausibly indicated a notion of homogeneity discovered across the subpopulation of RA and CAD patients. Our results also indicated that heterogeneous subpopulations in both RA (*p*-value = 0.044) and CAD (*p*-value = 0.015) were under-expressed compared to their controls (Figures 5H,I). The analysis was validated using GEO2R (42) differential expression analysis in GSE15573 (Figure 5J).

**FIGURE 6:**
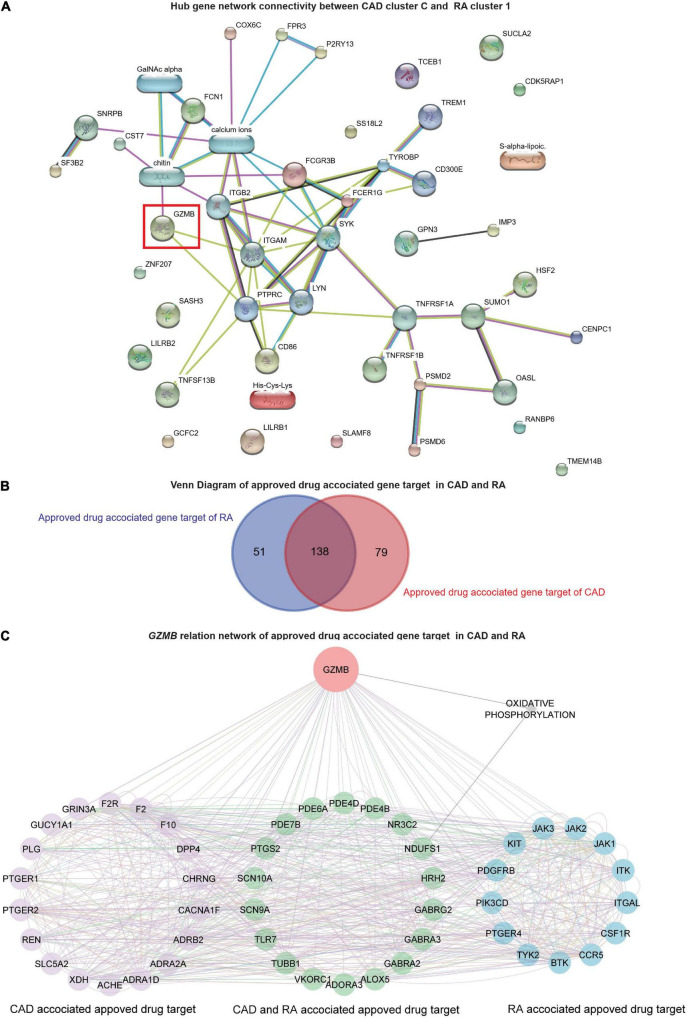
Network analysis and *GZMB*-related drugs targets. **(A)** The network analysis for the hub genes from cluster C of GSE59867 (CAD) and cluster 1 of GSE93272 (RA) hub genes using the STITCH database. The large nodes represent the known protein structures, wherein the small nodes represent the unknown protein structures. The identified subpopulation-differentiation marker, GZMB, was marked within a red box. The colors of the nodes and edges were predefined by STITCH. **(B)** Using the Open Target platform, the Venn diagram for the extracted drug-target genes (DTGs) of CAD and RA (blue). **(C)** Network-level association of *GZMB* and the related DTGs. The gene *GZMB*, the ReDisX identified gene of interest, the CAD-associated approved drug targets (purple), the approved drug targets for CAD and RA (green), and the approved drug targets for RA (blue) share a direct correlation. GeneMANIA predefined the colors of the edges.

Further, the STITCH analysis indicated a strong inter-relationship among the total identified 40 hub genes (20 hub genes each) from both the diseases, cluster 1 of RA and cluster C of CAD (Figure 6A). The STITCH-based interactions were derived from text mining, experiments, databases, co-expression, neighborhood, gene fusion, co-occurrence, and prediction with a medium confidence level (0.4). To further validate our result in whole blood tissue and the extent of the applicability in RNA-seq, we specified the interaction to be constructed from the whole blood RNA-seq data retrieved from the Expression Atlas using the URL, https://www.ebi.ac.uk/gxa/baseline/experiments (57) with medium confidence level (0.4). It suggested a prominent connected component, GZMB, and some discrete nodes such as SASH3 and SLAMF8 (Figure 6A).

### *GZMB*-related drugs target gene networks across coronary artery disease and rheumatoid arthritis

To evaluate the association of *GZMB* and its potential association with the drug target genes across CAD and RA, we employed the Open Targets platform (48). It facilitated the discovery of the available approved drugs and their association with the target genes (DTGs) of the diseases of interest, such as CAD and RA. A total of 189 DTGs for RA and 217 DTGs for CAD were identified (Figure 6B). The Venn diagram analysis indicated that 73.02% of the RA-related DTGs intersected with CAD-related DTGs (Figure 6B). On the other hand, 63.59% of the CAD-related DTGs intersected with RA-related DTGs (Figure 6B). The detailed gene list is available in Supplementary Material 4.

To investigate the network-level association of *GZMB* and those 268 unique DTGs (as shown in Figure 6B), GeneMANIA (36) was employed. The considered interactions were consolidated pathways, wiki-pathway, reactome, co-expression, physical interaction, drug-interaction, predicted, co-localization, pathway, shared protein domains, and genetic interaction. This analysis extracted a total number of 36 additional genes out of the GeneMANIA knowledge base. Altogether it returned a total number of 305 genes and 18,138 interactions within the reconstructed network (Supplementary Material 5). Further, we have narrowed our analyses by focusing on the *GZMB*-related DTGs in CAD and RA. It produced a network with the selected DTGs sharing direct interaction with *GZMB* (Figure 6C). It constructed the network containing a total number of 51 genes and 755 interactions (Figure 6C).

## Discussion

Diagnosis of a disease is used to get a universal consideration across the patients. Identifying the clinicopathological variables associated with the patients looks straightforward, but it is one of the trickiest and most sensitive concerns to be precisely addressed in terms of physiological and clinical perspectives. The current practices of classifying clinicopathological variables among the patients, also referred to as classifications of diseases, are typically relied on medical investigations such as physical and visual examinations, estimation of biochemical parameters, medical imaging methods, and of late, a few genomic-markers-based diagnostic approaches, which are although not widely and commonly practiced. Often the genomic disparity among the individual gets overlooked. It is something like a one-size-fits-all concept. However, with the advancement of the data-driven healthcare revolution and leveraging the high-throughput omics data, we could further investigate the issue as mentioned earlier to have better resolution in assessing the clinical conditions rather than a “typically classified disease and its same related treatments for all” for each individual. It may offer us a clue to identify the real root cause at the molecular layer and personalized or customized treatment plans. Our study supported our core rationale by introducing a functional concept of redefining diseases (with the working example of RA and CAD datasets, GSE93272 and GSE59867, respectively) based on individualized molecular characteristics using the ReDisX framework. Interestingly, our results indicated some exclusive subcategories featuring distinct molecular-genetic signatures within the “conventionally classified” disease category. ReDisX has tried identifying those distinct subpopulations upon thoroughly analyzing those signatures to reassign their clinical category focusing on precise identification.

### Rationale, and quality assessment of ReDisX performance on the selected datasets

#### The rationale of using blood-based transcriptomic data to deploy ReDisX

We used two disease conditions, RA and CAD, in our study. RA is diagnosed mainly by blood-based examination or synovial tissue examination. And CAD is typically diagnosed by CT-imaging techniques supported by blood-based examinations. In both cases, specific markers are expected and usually found to be elevated or depleted in the patients. Analyzing the blood from the patients should report those dysregulations of the blood-based factors instituted through their corresponding transcriptional mechanisms. Hence, analyzing the blood-based transcriptome must aid in reporting those anomalies. It could be argued that high-throughput gene expression analysis of specific cell types or tissue subsets could theoretically have been more informative than whole blood and peripheral blood transcriptome analysis (58, 59).

Nevertheless, available methods for subset-specific expression profiling are not quite adequate for extensive studies and the choice of the cell types to be analyzed is also not so determined. A certain extent of population-dependent effects is unavoidable (58). Recently, it has been strongly endorsed that blood-based transcriptome analyses are more efficient in capturing the global gene expression landscape and can be served to facilitate the detection of deregulated genes or gene products. Whole blood transcriptome can significantly predict tissue-specific expression levels for ∼60% of the genes on average across 32 tissues (60). The tissue-specific expressions inferred from the blood transcriptome are almost as good as the measured tissue expression in predicting disease state in many complex disorders (60). It is advantageous in two significant ways; one, it is sufficient to capture the global gene expression landscape much earlier and can serve as a well-accepted reporter for clinical conditions of the patients, and two, it is minimally invasive, more generalized, and technically less complicated. It also overcomes the fixed snapshot of localized tissue-specific sampling with a time stamp and offers a more dynamic global snapshot of the clinical conditions of an individual.

In our study, the analyses and validation of hub gene identification were not varied across blood and synovial tissue samples. To identify the core hub genes for RA, we employed ReDisX using a blood-based test dataset, GSE93272, and validated the results with two other validation datasets obtained from blood and synovial tissue, GSE15573 and GSE77298, respectively. In all cases, the results were consistent (Figure 5A and Supplementary Material 3). So, we may suggest that ReDisX-discovered marker hub genes for RA were well discernable from the blood transcriptome. Our method typically intends to devise a minimalistic approach yet high yield precision performance. High-quality RNA can also be extracted from blood samples. It is beneficial for extensive population studies while reducing technical and source variability that may limit the reproducibility of results and introduce a systematic bias in multicenter studies. Thus, for the long-term objective-wise, our study is more suitable in the context of easy-to-use and quick support before undergoing a confirmatory diagnosis for receiving further treatment. Aligning this motivation, the scope of blood-based transcriptome analyses should be more suitable.

#### The quality assurance of ReDisX performance

We have used two datasets, GSE93272 (retrieved from whole blood gene expression of RA patients) and GSE59867 (retrieved from peripheral blood samples of CAD patients), as the primary datasets for testing on which the performance of ReDisX been reported. Two other datasets, GSE15573 (retrieved from peripheral blood samples of RA patients) and GSE77298 (synovial biopsies of RA patients and healthy controls), were used for statistical validation purposes. It was to indicate that ReDisX output did not interfere with the origin of sampling. The discovery of hub genes was consistent across all datasets. Further, we have performed an additional validation study for the ReDisX-discovered hub genes on the validation dataset, GSE23561. We have chosen this dataset as it contains peripheral blood gene expression profiles for both the concerned diseases, RA and CAD. The representation of the datasets is shown in Table 1.

Our additional dataset analysis also exhibited consistency with the earlier ReDisX-discovered hub genes, including identifying *GZMB* as a predictive subpopulation differentiation marker for both RA and CAD populations (Figure 3A). The performance of ReDisX on the gene expression profiles for RA and CAD from the dataset GSE23561 showed the consistent discovery of hub genes as did earlier within cluster C of the CAD dataset, GSE59867. Next, it re-assigned the patients with a new label. Figure 3A indicates the cross-dataset validation of ReDisX performance to identify the hub genes from GSE23561. The RA and CAD patients were reassigned into new groups. Group 1 (blue) indicated the inclusion of RA and CAD patients as they were presumed to consist of similar hub genes despite being labeled differently by the conventional diagnosis as initially mentioned in the dataset, GSE23561. Group 2 (red) includes only a group of patients labeled as CAD in the dataset. ReDisX was applied to the dataset for all the patients and distinguished two unique groups that varied from the original classification. This new stratification of patients was based on discovering some significant hub genes that may function as subpopulation differentiation markers. Hence, we may assume that ReDisX-discovered hub genes are not dataset-specific; instead, they can be widely deployed for disease cases. It may also indicate the generosity of the ReDisX framework. A GEO2R analysis relying on the ReDisX-based labels in GSE23561 asserted the validation of the *GZMB* as a subpopulation differentiation marker as discovered by ReDisX. *GZMB* was statistically significant as a subpopulation differentiation marker at *p*-value < 0.05 and | log-fc| > 1 (Supplementary Material 6). So, this additional analysis supports the generosity of ReDisX-discovered hub genes and the importance of *GZMB* as hypothesized.

### ReDisX offers better precision and personalized diagnosis strategy

Diagnosis of diseases usually relies on biochemical and pathological assessment, imaging techniques, and molecular analyses (61). In many instances, especially for complex diseases, it is inadequate to explain the heterogeneity among the patients within the same disease and homogeneity across the patients from different diseases (5, 6, 13). Therefore, redefining the diseases to elucidate the heterogeneity within a disease and homogeneity across the diseases will significantly ensure precision diagnosis and personalized treatments (62, 63). So, it ensures precision and a personalized model of identifying patients’ clinicopathological conditions. Many computational and experimental studies have evolved in this avenue (3, 4, 22). They mostly attempted to address the heterogeneity within the patients under the same disease and homogeneity across the patients from different diseases. However, an interesting question about the extent of homogeneity amidst the heterogenous sub-population of different diseases remains unexplored. The consequent similarities and dissimilarities at the molecular level have not yet gained comprehensive attention. Therefore, in this study, we have introduced ReDisX, a robust, scalable, and pathologically relevant computational framework to characterize the patients based on specific molecular-genetic signatures. We have systematically deployed the ReDisX framework considering two disease cases, RA and CAD. We have analyzed their transcriptional profiles to characterize the pathological similarity of their subpopulation. In the future, it perhaps guides us to extend this foundation to other diseases to redefine their clinicopathological status aiming toward precision diagnosis and identifying personalized targets for therapeutic interventions.

Our proposed framework, ReDisX, could differentiate the disease heterogeneity among the patients by optimally clustering them based on individual gene expression profiles. One of our past studies indicated that RA and CAD used to share inflammatory pathways (13), and both of them consist of several subtypes (6, 64). However, the detailed characteristics of the subpopulations of both RA and CAD were not yet fully explored. Our data suggested that the ReDisX could identify a distinct subpopulation in RA and CAD that were susceptible to mispronounced by the standard diagnosis criteria (as the data was captured originally at the point of diagnosis) without the ReDisX-based recategorization (Figures 2A,B, 4A,B). On the other hand, our results also indicated an identified homogeneity across the subpopulation of CAD to the RA patients (Figures 5, 6A). These characterizations were also validated using two other validation datasets, strengthening our hypotheses (Figure 5A and Supplementary Material 3). The heterogeneity mentioned above and homogeneity were not identified with the original diagnosis strategy mentioned in the dataset features. Therefore, the conventional modes of diagnosing diseases and categorizing the patients overlook certain minute discrepancies at the individual molecular-genetic signature level. It may indicate a potential cause for failure of diagnosis of those specific subpopulations of patients; therefore, their receiving treatments might induce either inefficiency or some adverse effects. In many acute or severe clinical conditions, such inadequacy in characterizing the actual cause might significantly delay the treatment process or prognostic outcomes. On this verge, our proposed framework, ReDisX, showed an enhanced efficiency in accessing minute details of the molecular signatures in characterizing the patients.

### ReDisX supports advancing the screening of precise druggable target genes

Based on the ReDisX framework, our results suggested that *GZMB* was under-expressed in the subpopulation of both the diseases RA and CAD (Figure 5). A study by Joehanes, R. et al. concluded that CAD patients with *GZMB* expressions exhibited a significant negative fold change (FDR < 0.05) compared to their healthy controls (25). Although there was no solid evidence showing the direct pathological relationship between the *GZMB* under expression and the occurrence of CAD and RA, we have identified some diseases linked to *GZMB and* correlated with CAD and RA. For example, CAD was found to be correlated with Moyamoya Disease (MMD) (65) and pneumonia (66). Furthermore, Xing Peng et al. discovered that MMD patients with downregulated *GZMB* were also enriched with the downregulated genes of CAD (65). MMD is defined as an occlusive intracranial arteriopathy with abnormal cerebral vascular collateral networks and is primarily known to involve intracranial arteries, but systemic arterial involvement, especially endothelial hyperplasia, was also reported (67). Cases of stenoses in the left coronary artery and renal arteries were reported, which were further confirmed with the arterio-angiography examination (68). The association between coronary heart disease and MMD has been studied in other research. Many studies suggested having common etiological factors between CAD and MMD (68–78).

Interestingly, a recent case report by James Livesay and Jeffrey Johnson indicated a direct incidence of STEMI in an MMD patient. Perhaps, to the best of our knowledge, this is one of the first-in-class direct case reports of CAD in a Caucasian female with reported MMD, presenting with cerebral vasculature complications (79). They emphasized a critical notion that extracranial vascular complications in MMD patients are rare but can significantly impact a patient’s overall morbidity and mortality. Thus, clinicians should be aware of the cardiovascular complications, including the coronary plaque progression seen in MMD patients, and assess for adequate medical management. Another contemporary study by Peng et al. (79) stated that CAD used to share many clinical symptoms with MMD, including progressive narrowing or occlusion of involved arteries. MMD has emerged partially analogous to CAD on the peripheral blood transcriptomic level plausibly due to the underlying inflammations (79). In addition, cases of CAD in patients with MMD have been extensively reported (65, 72, 77, 79, 80). It may indicate that MMD is a systemic vasculopathy wherein both the circulating inflammatory factors and the imbalance of cell populations in the blood are responsible for the progressive narrowing or occlusion of the involved arteries. MMD could likely lead to occlusion of coronary arteries through certain systemic etiologic factors in the blood. Many other studies also supported a correlation between MMD and CAD (67, 79).

Now, concerning the RA cases, a recent report showed that a patient with a history of RA for 15 years also suffered from cerebral rheumatoid vasculitis (81). A meta-analysis of 23 studies found that the patients with RA shared a higher risk (∼1.68 times) of hemorrhagic stroke than normal individuals (82). It suggested that underexpression of *GZMB* may be a risk factor for RA patients developing MMD, but further investigation should be conducted to support this hypothesis. It also intrigued a sense of investigating *GZMB* as a potential drug target, especially for the niche subpopulation identified across the RA (cluster 1) and CAD (cluster C) patients wherein *GZMB* was under-expressed. Consequently, our results suggest that the patient-specific individual differential expression features of *GZMB* may explain the plausible underlying inflammatory mechanisms across those clinical conditions.

Studies also showed that excessive *GZMB* is related to inflammation (83), a common pathological characteristic of CAD and RA (84–86). However, those investigations did not consider minute fluctuations observed within some subpopulations of the CAD and RA patients who significantly exhibited *GZMB* under expression. Interestingly, our results characterized those distinct subpopulations of both CAD and RA as having *GZMB* under expression (Figures 5H,I). Our study validated this claim with another RA dataset (Figure 5J). The study by Joehanes R. et al. supported this indication (25). The inhibition strategy for *GZMB* overexpression cases was considered a new therapeutic target for CAD and RA. Yue Shen et al. demonstrated that the under-expression of *GZMB* protected against Ang II-induced cardiac hypertrophy and cardiac fibrosis, microhemorrhage, inflammation, and fibroblast accumulation (85). Cui-Xia Bao et al. show that *GZMB* gene silencing inhibits the MAPK signaling pathway by regulating the expressions of inflammatory factors (55). Inherently, this demands our attention to investigate further those subpopulations of *GZMB* overexpression and underexpression and associated different clinicopathological statuses. After distinguishing its expression profiles within the subpopulation, it also sparked an idea to differentially employ *GZMB* as a drug target for personalized therapeutic intervention. Altogether, it essentially re-emphasizes that it is necessary to redefine the disease to navigate the hidden heterogeneity and cross-disease homogeneity to offer better precise categorization, diagnosis, and treatment for the patients.

### ReDisX endorses the *GZMB* as a robust prognostic subpopulation differentiation marker to devise a personalized druggable target

GZMB (granzyme B) is a serine protease encoded by the *GZMB* and is commonly found in the natural killer (NK) cells and cytotoxic T cells (CTLs) (87). It has been reported to participate in different inflammatory signaling pathways such as inducing cell death, apoptosis (88), and suppression of viral replication (89). It has also been reported as a drug-gene target for tumor therapy (90), especially with cisplatin (39) and mannose (39). It is also reported that GZMB injures endothelial cells in patients with immunological dysregulation, such as recipients of allograft transplantation. It could be of viral origin or any other exogenous factors. It is a deep concern, and there is enough evidence claiming that direction especially discussing the role of GZMB in acute coronary syndrome after heart transplantation wherein the acute graph versus host disease driven by donor T cells and an inflammatory cytokine storm could be expected, which results in target tissue destruction via apoptosis (91–93).

However, on the other side, several studies have strongly suggested the link between GZMB and atherogenesis, which is one of the primary mechanisms contributing to developing CAD in patients without any reported transplantation and/or involvement of donor T-cells. For example, Chamberlain, C., and Granville, D., suggested that in atherosclerosis, immune-mediated cellular apoptosis plays a crucial role, and GZMB interferes there (94). In their study, CAD patients were defined as patients showing significant coronary stenosis diagnosed by selective coronary angiography. The authors excluded patients with acute coronary syndrome, acute inflammatory disease, acute renal failure, hematological disorder, malignancy, and patients taking immunosuppressive medicine (94). The authors reinstated that the perforin/granzyme system usually induces apoptosis of infected cells and cancer cells.

Conversely, they also emphasized that plasma GZMB was an independent factor for the severity of CAD. On the other hand, the administration of serine protease inhibitor was reported to attenuate atheromatous plaque formation in apoE-knockout mice (95) and vascular injury after allograft transplantation (96). So, serine proteases (like GZMB) may play an essential role in atheromatous plaque formation in certain patients. In another study, Saito, Y et al. indicated that GZMB induces apoptotic cell death and degrades the extracellular matrix, weakening the fibrous cap of atheromatous plaques (97).

However, there is no clinically recognized universal baseline for the *GZMB* expression pattern within the same or different diseases. Overexpression of *GZMB* was found to promote CAD (98, 99), wherein under-expressed *GZMB* was correlated with CAD (25) pathophysiology. It is a contradictory role of *GZMB* in CAD. Similar contradictory functions of *GZMB* were also reported in RA patients. For instance, the up-regulated *GZMB* was reported as an indicator of inflammatory diseases such as RA (83), and in another research, the downregulation of *GZMB* by shRNA-mediated silencing was shown to promote RA in a rat model (55). It was found that *GZMB* silencing inhibited the MAPK signaling pathway by interfering with the expression of several inflammatory factors such as bcl-2, caspase, and certain angiogenic factors such as VEGF and bFGF. Indeed, a gene and its contradictory behaviors instigate an ambiguity in prognosis and consider that candidate for therapeutic development. It induces a layer of obscurity about devising inhibitors or activators to modulate *GZMB* within the same diseases and for different diseases, such as RA and CAD. Such complications represent the lack of understanding of the heterogeneity of *GZMB* across the subpopulations. Another recent single-cell study has also shared a similar view to consider the clinical identifications of RA based on different subsets of markers and reported the potential of *GZMB* as a subpopulation differentiation marker (100). So, it implies a solid motivation to utilize this gene, *GZMB*, as a prognostic marker or therapeutic target on a personalized basis. Added to their findings, determining the allocation of those markers across the RA patients at a personalized level would undoubtedly be an essential direction. The extent of the variations and discrepancies in their expression patterns prevails across the distinct subpopulation of RA patients is a prominent study scope. It will be even more interesting to navigate such similarities across the subpopulation of different diseases or dissimilarities within the same disease. Interestingly, our proposed framework, ReDisX, addresses the expression pattern variation of *GZMB* across the different subpopulations of patients.

#### ReDisX performance was not influenced by the drug treatment in the rheumatoid arthritis dataset, GSE93272

*GZMB* was found to be upregulated among the RA patients receiving the drug treatments but not for the other RA patients without drug treatments. However, in some subpopulations of RA patients where *GZMB* was found to be downregulated even after the drug treatments. Those specific cases were merely not attended by the drug-labeled-based classification alone. However, with the ReDisX, we could identify those subpopulations distinctly within the RA dataset. To connect the clinical impact, we may presume (hypothetically) that if any therapy is targeted for *GZMB* to manage the RA cases, that distinct subpopulation may not respond adequately to that specific treatment. For example, in the RA dataset, some patients with *GZMB* downregulation used to receive drug treatments such as IFX.IR, MTX, MTX.IR as per the study description.

Nevertheless, they also share a similar extent of GZMB expression as other RA patients do without receiving any treatments. So, it may indicate that those subpopulations of RA patients receive either the wrong or inadequate treatments. Eventually, the RA patients without receiving drug treatments showed non-significant downregulation of *GZMB* (at adjusted *P*-value 7.438697e-01 by ANOVA) (Supplementary Material 7), as our additional box-plot analysis indicated (Figure 3E). On the other hand, ReDisX discovered a subpopulation within RA that shows statistically significant downregulation of *GZMB*. So, ReDisX helps to provide additional information on the same dataset wherein the drug treatment labeling is limited to indicate that precision information. With the evidence from both aspects of GZMB, we may pursue that GZMB may be involved in forming atherosclerosis in a group or subgroup of patients with immunological disorders. In our study, we included RA patients, which are usually reported to have an immunological imbalance. Hence, GZMB may function as an essential mediator to connect RA and the emergence of coronary atherosclerosis in the subgroup of RA patients. Thus, the role of GZMB can be established as a predictive subpopulation differentiation marker.

In our study, the ReDisX framework validates the potential of *GZMB* as a subpopulation differentiation marker within the same disease, e.g., under expression of *GZMB* in cluster 1 of RA patients and overexpression of *GZMB* in clusters 2 and 3 of RA patients. Furthermore, it also demonstrated the homogeneity of *GZMB* expression across different diseases. For example, the under-expression of *GZMB* in cluster 1 of RA patients shared a similar expression pattern with cluster C of CAD patients, further supported by the functional enrichment analysis. These disparities strengthen the indication of the sensitivity of the strategies for therapeutically modulating *GZMB* expressions. Thus, it is highly recommended not to use a *GZMB* inhibitor/activator for all the patients suffering from RA and CAD, respectively.

A study reported the generic use of *GZMB* inhibitors to manage RA (55). However, our study flags a concern here and suggests the use of *GZMB* inhibitor to be devised on a personalized basis upon evaluating its specific patterns within the patient subpopulation. Similarly, it can also apply to CAD patients too. Additionally, the ReDisX framework suggests the specific drug designated for a subpopulation of RA patients with *GZMB* under-expression can be repurposed for the subpopulation of CAD patients having *GZMB* under-expression but never be used for the subpopulation of RA patients with *GZMB* overexpression. Hence, ReDisX demonstrated a data-driven ability to designate the *GZMB* as a potent subpopulation differentiation marker for RA and CAD. It also indicated a way to precisely deploy them for therapeutic development and a plausible strategy to repurpose those therapeutics backed by personalized gene-expression data.

### ReDisX suggests *GZMB* as a strategic focus for drug repurposing

*GZMB* inhibitors have been reported as therapeutics to manage inflammations related to RA (55) and CAD (97, 101). However, the contradictory dual role of *GZMB* has made the discovery process and its clinical application ambiguous (102). Studies have reported that *GZMB* overexpression and underexpression are linked to different clinical conditions, such as RA, CAD, and MMD (55, 65, 97). It certainly makes the strategic development of therapeutics and/or discovering drugs against *GZMB* challenging. On the other hand, *GZMB* has been repeatedly endorsed as a prominent prognostic marker for many inflammatory pathways, especially related to RA, CAD, and angiogenesis (55). Hence, having a straightforward strategy to deal with *GZMB* is essential. ReDisX-based indication on *GZMB* and its prominent role as a prognostic marker has been further strengthened by the Open Target analyses (Figure 6C). It intensifies that the ReDisX-based identification is not only computationally validated; instead, it has a solid connection to be a potential personalized druggable target. It has also gained scientific support from different experimental studies and a presumed knowledge base (see “Results” Section for details). It motivates us to devise a strategy to deal with those ambiguities. So, ReDisX perhaps offers a plausible solution to designate the discrepancies of *GZMB* to be deployed as a prognostic marker and a target for therapeutic development.

### ReDisX is the robust, scalable, reproducible framework

ReDisX characterizes heterogeneous subpopulations within a disease and homogenous subpopulations across different diseases. This study used RA and CAD as examples of stretching in connection with one of our prior studies (13). We have established our ReDisX framework as a proof of concept. It is scalable and can be deployed in other disease cases, provided the input data are in the same format (as described in Section “Materials and methods”). In this study, the considered dataset for CAD represented STEMI which is a large vessel disease unlike typical cases of RA. However, deploying our ReDisX framework and demonstrating its functions requires at least two datasets belonging to the different diseases labeled by the conventional diagnosis with a certain extent of the pathophysiological connection between them. And our primary investigative motivation was to distinguish the genomic signature from the personalized genomic data aiming to differentiate one patient from another and/or a patient subgroup from another subgroup despite being in the same and/or a different group as conventionally labeled. It also indicated patient-specific similarity in genomic signatures across two disease groups as typically labeled. It strengthens further a direction of investigation to identify certain shared features such as common drug targets or any lead drug candidate to be repurposed for a specific group of individuals. Surprisingly, both diseases seem far apart from the vascular anatomy angle, but several studies indicated their strong relationship regarding pathophysiological influences. For instance, in 2021, in a review published in The Lancet Rheumatology (103), Hansildaar R et al. indicated the occurrence of rheumatoid arthritis (RA) as a strong independent cardiovascular risk factor. Patients with RA were reported to have a 2X higher risk of developing atherosclerotic cardiovascular disease than the general population. Our concerned CAD dataset certainly belongs to this category of cardiovascular diseases. The authors also added that despite sharing completely different pathogenesis, the underlying pathophysiological mechanisms in systemic inflammation might overlap to a certain extent. Following the recognition that systemic inflammation has a critical causative role in cardiovascular disease, anti-inflammatory therapy in both conditions and urate-lowering therapies in gout are expected to lower the cardiovascular burden of patients. Several other studies also reported the role of inflammation and its mediators in atherosclerosis plaque development to promote CAD and suspected some of those inflammatory pathways might share a connection to contribute to the systemic inflammatory status in RA (104–106). So, the basis of different vascular architectural manifestations for RA and CAD may not affect the rationale for our study or the performance of ReDisX. The selection of datasets was independent of those minute anatomical considerations as it only aims to classify the conventionally labeled patient populations (RA and CAD, for example, used in this study) by ReDisX-discovered genomic signature. Methodologically, the infrastructure of ReDisX considers the ward’s distance in Hierarchical clustering and graph connectivity of the CMF model, wherein the ward’s distance is an established formula, and the hyperparameters in the CMF model were chosen as stated in the original paper (23, 24). Hence, the reproducibility of the ReDisX could be ensured as long as the pre-labeling seeds are consistent.

The current molecular pathological studies practice analyzes the tissue-of-origin for any diseases (107). For example, the diagnosis of RA is typically made from synovial tissues (108), wherein cancers are diagnosed from tissue biopsies in the case of solid tumors (109). However, liquid biopsies are widely practiced (110). Eventually, the sampling procedures within the clinical setup gradually incline toward non-invasive or minimally invasive ways while capturing the maximum information of the concerned underlying pathological conditions (110). So, keeping that vision, we put our effort into establishing our model with the blood sample, which is comparatively less invasive (26, 27, 40) and more accessible to be retrieved from patients, and holds the potential to be a more expansive repertoire of the clinicopathological as well as molecular genetic conditions or biomarkers. However, based on our study objectives, we need to choose a suitable tissue to analyze all diseases. Some potential issues such as whole blood (111, 112) and the gut microbiome (113), are suitable for our study. The ReDisX framework could be extended and improved in the future using multimodal data besides mRNA expressions such as methylation and miRNA interference.

## Conclusion

ReDisX demonstrates a scalable data-driven framework to characterize the genomic signature uniquely and redefines the disease diagnosis strategy. It indicates a high-resolution precision and personalized diagnosis. It logically distinguishes the subpopulation heterogeneity within a disease and homogeneity across different diseases. It supports the personalized screening of DTGs. Our study with RA and CAD explains its efficiency in characterizing a subpopulation differentiation marker, *GZMB* augmenting it as a potential personalized druggable target. Discovering the RA-characteristics-dominant CAD subpopulation supports one of our primary intentions to redefine the disease diagnosis with a personalized molecular signature. It offers a new insight to understand the disease and revise our consecutive treatment plans. In addition, this study also suggests *GZMB* as a strategic focus for drug repurposing.

ReDisX framework is scalable and methodologically flexible to be further improved. We desire to deploy it to other complex disease cases and enhance the core algorithms by incorporating multimodal data. However, the clinical prospects of disease redefinition by ReDisX are yet to be validated in terms of the quality of precision diagnosis and efficacy of the recommended repurposed drug candidates against the indicated clinical conditions. Last, it elucidated a novel perspective to rethink diagnosing diseases and the emergence of personalized therapeutic development.

## Strengths of our study

1. ReDisX framework is scalable and can be adapted to different biomedical applications.
2. It is a first-in-class CMF-based ML algorithm that precisely discovers signature gene expression features from the given patient data, and also, the small sample size does not affect the accuracy.
3. It discovers distinct heterogeneous subpopulations within a disease and homogenous subpopulations across different diseases.
4. It offers a clue to discovering new therapeutic targets and drug repurposing.
5. It can adopt a small sample size and return accurate predictions, consistent with many experimental studies.

## Limitation of our study

1. Hyperparameters used in ReDisX are data sensitive. It demands fine-tuning for different input data from different diseases.
2. Removal of cross-platform batch effect is susceptible to affect the accuracy of the analyses. Thus, it may require very careful preprocessing of input data.
3. The study’s current proposition, including mRNA gene expression data, could partially explain the body’s underlying mechanism.
4. The proposed framework of ReDisX is based on a single omics analysis algorithm, and its impact in the case of multi-omics input across multi-tissue data is not optimized yet.
5. The generalization of our proposed method is still subjected to be validated before any clinical applications.

## Data availability statement

Publicly available datasets were analyzed in this study. This data can be found here: GSE93272 (https://www.ncbi.nlm.nih.gov/geo/query/acc.cgi?acc=GSE93272) and GSE59867 (https://www.ncbi.nlm.nih.gov/geo/query/acc.cgi?acc=GSE59867). Validation datasets are GSE15573 (https://www.ncbi.nlm.nih.gov/geo/query/acc.cgi?acc=GSE15573), GSE77298 (https://www.ncbi.nlm.nih.gov/geo/query/acc.cgi?acc=GSE77298) and GSE23561 (https://www.ncbi.nlm.nih.gov/geo/query/acc.cgi?acc=GSE23561). The code is deposited in (https://github.com/YIPhiufung1997/ReDisX).

## Author contributions

HY and DC contributed to the conceptualization, contextualizing, study designing, refining, analyzed and interpreted the data, and also prepared the original draft and the figures of the manuscript. DC contributed to the overseeing the project and revising the manuscript. XT, KL, and CZ contributed to the constructing the algorithms and mathematical executions. KW, YL, YG, LL, and DG contributed to the data interpretation. DG also supported the work. HZ, XT, and AL supervised and supported the work. All authors provided critical feedback and approved the submitted version.

## Footnotes

1
http://stitch.embl.de/

2
https://www.opentargets.org/
