# Supplementary material for: ReDisX, a machine learning approach, rationalizes rheumatoid arthritis and coronary artery disease patients uniquely upon identifying subpopulation differentiation markers from their genomic data
Source: Front Med (Lausanne). 2022 Aug 22;9:931860. doi: 10.3389/fmed.2022.931860 (PMC9441882; doi:10.3389/fmed.2022.931860)
Supplement: Supplementary file 2 [file Data_Sheet_2.ZIP › supplementary2/supplementary2.1_enrich_clusterC/GSEA_Disgenet_GSE59867_ReDisXclus3.pdf]

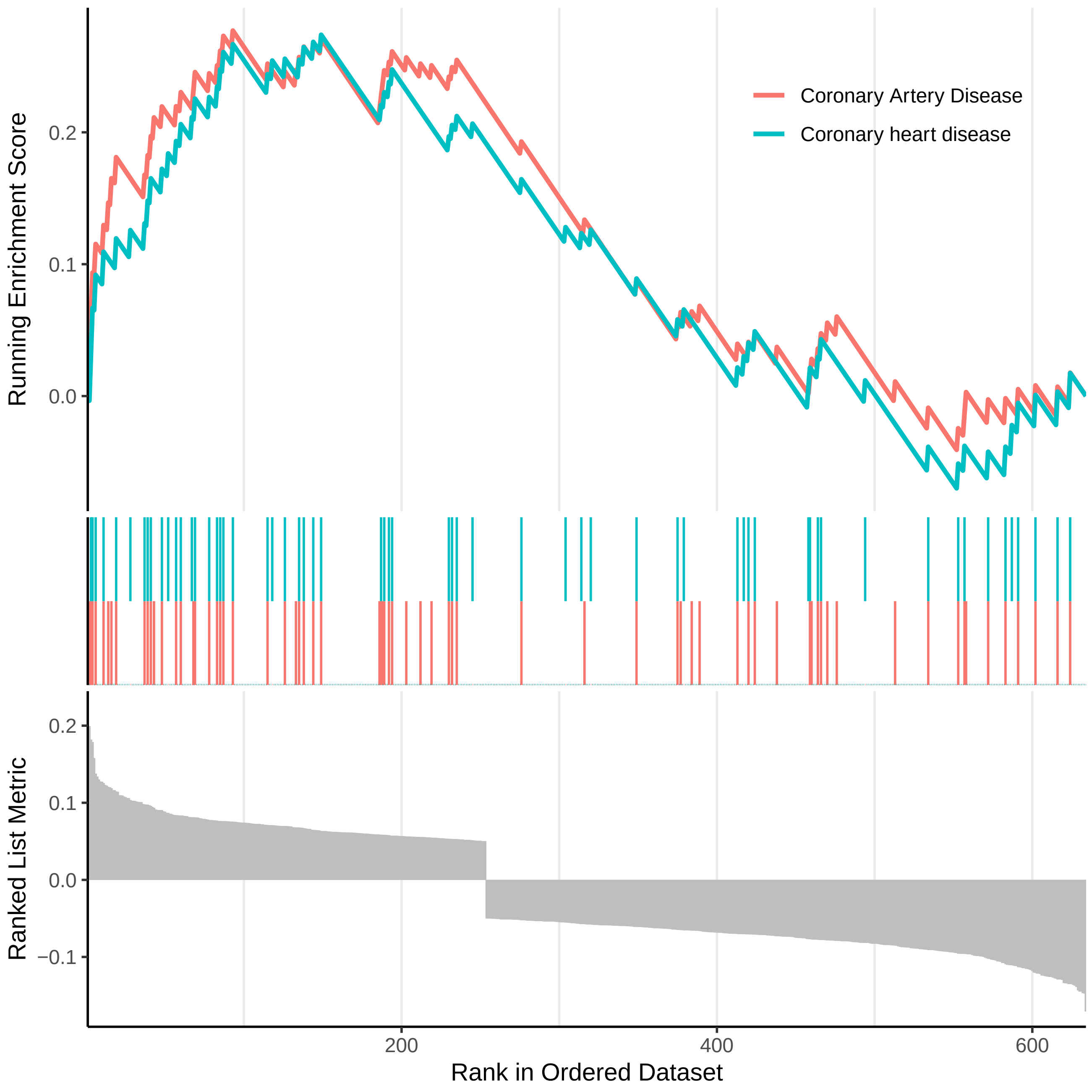

Coronary Artery Disease
Coronary heart disease
Running Enrichment Score
0.2
0.1
0.0
Ranked List Metric
0.2
0.1
0.0
−0.1
200
400
600
Rank in Ordered Dataset
