# Supplementary figures and images for: ReDisX, a machine learning approach, rationalizes rheumatoid arthritis and coronary artery disease patients uniquely upon identifying subpopulation differentiation markers from their genomic data
Source: Front Med (Lausanne). 2022 Aug 22;9:931860. doi: 10.3389/fmed.2022.931860 (PMC9441882; doi:10.3389/fmed.2022.931860)

# Enrichment analysis by Enrichr

Enriched terms

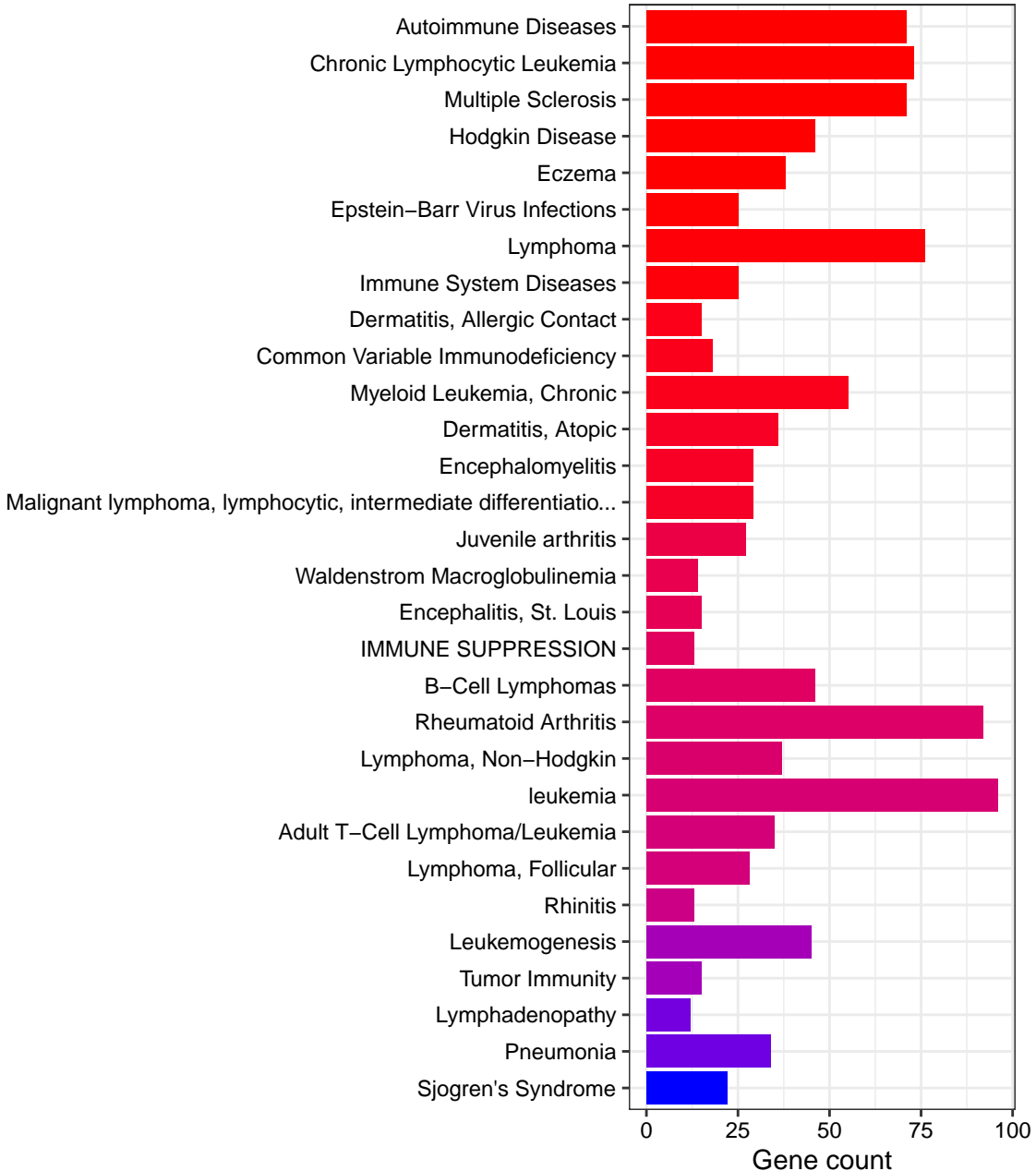

P value

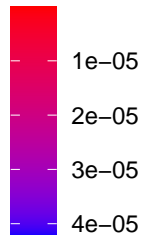

Supplement: Supplementary file 2 [file Data_Sheet_2.ZIP › supplementary2/supplementary2.1_enrich_clusterC/Barplot_ReDisX_GSE59867_clus3_DisGeNet.pdf]

# Enrichment analysis by Enrichr

Enriched terms

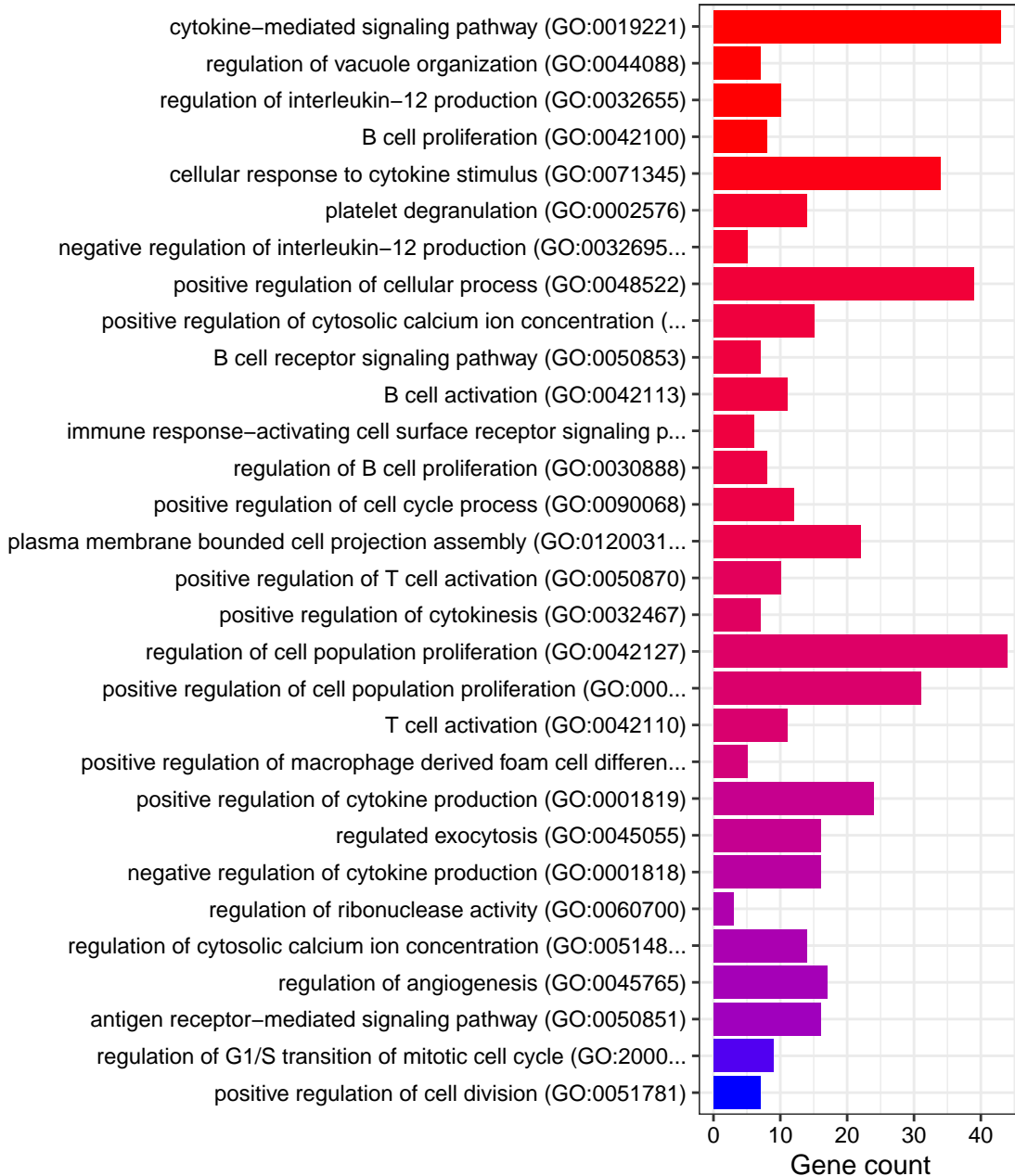

P value

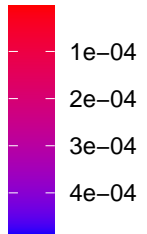

Supplement: Supplementary file 2 [file Data_Sheet_2.ZIP › supplementary2/supplementary2.1_enrich_clusterC/Barplot_ReDisX_GSE59867_clus3_GO_BP.pdf]

# Enrichment analysis by Enrichr

Enriched terms

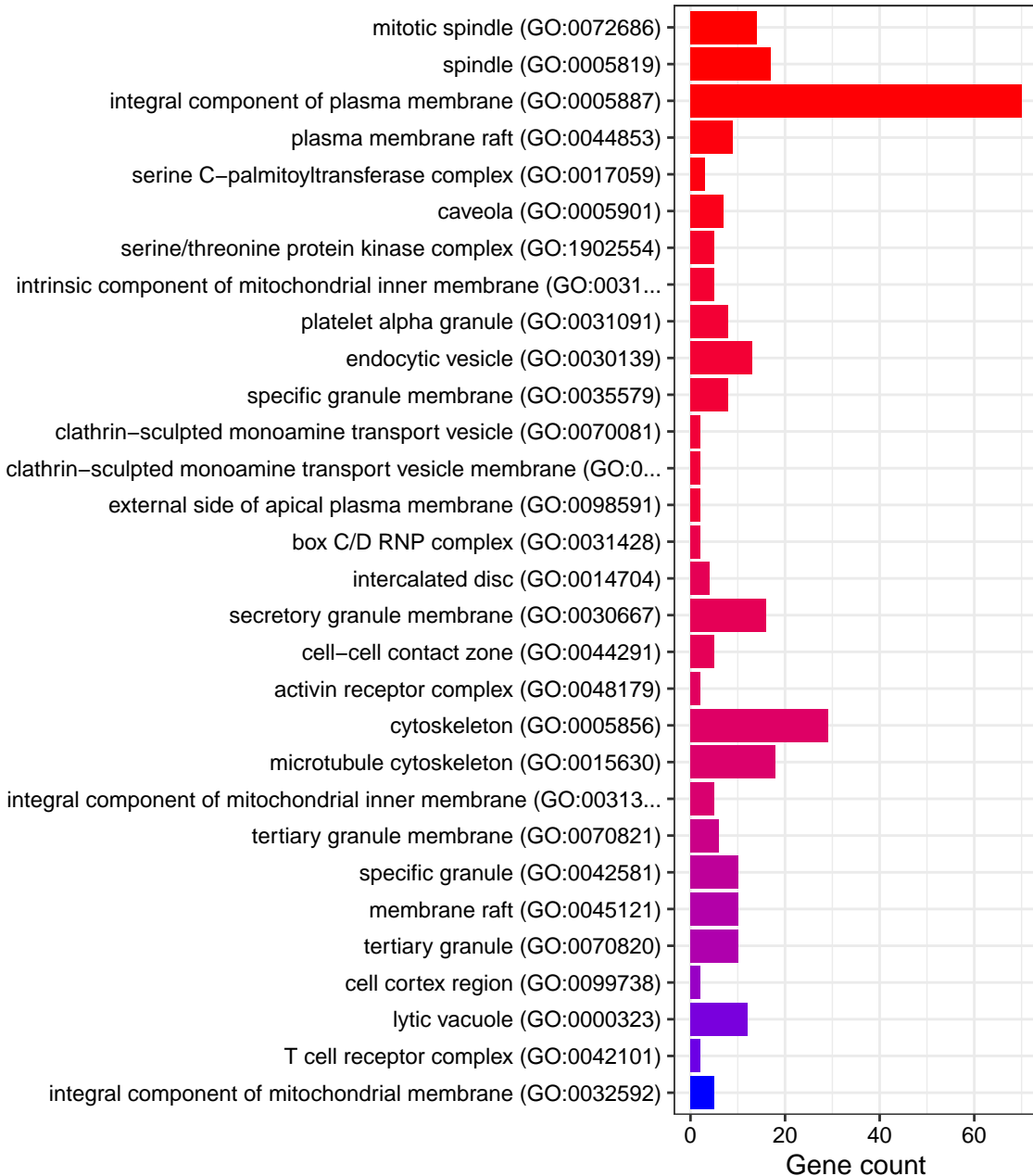

P value

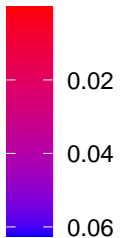

Supplement: Supplementary file 2 [file Data_Sheet_2.ZIP › supplementary2/supplementary2.1_enrich_clusterC/Barplot_ReDisX_GSE59867_clus3_GO_CC.pdf]

# Enrichment analysis by Enrichr

Enriched terms

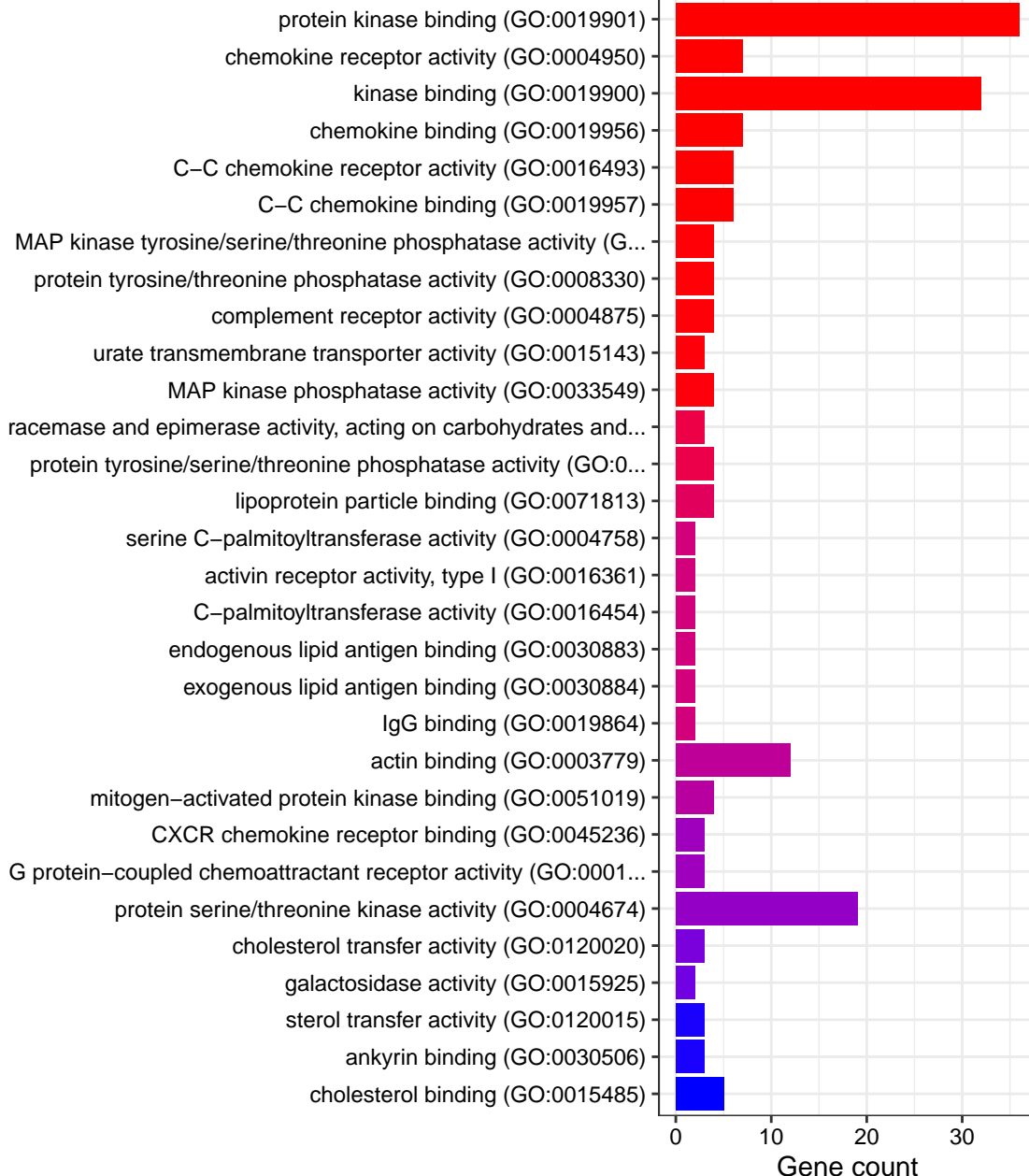

P value

0.005  
0.010  
0.015  
0.020

0 10 20 30  
Gene count

Supplement: Supplementary file 2 [file Data_Sheet_2.ZIP › supplementary2/supplementary2.1_enrich_clusterC/Barplot_ReDisX_GSE59867_clus3_GO_MF.pdf]

# Enrichment analysis by Enrichr

Enriched terms

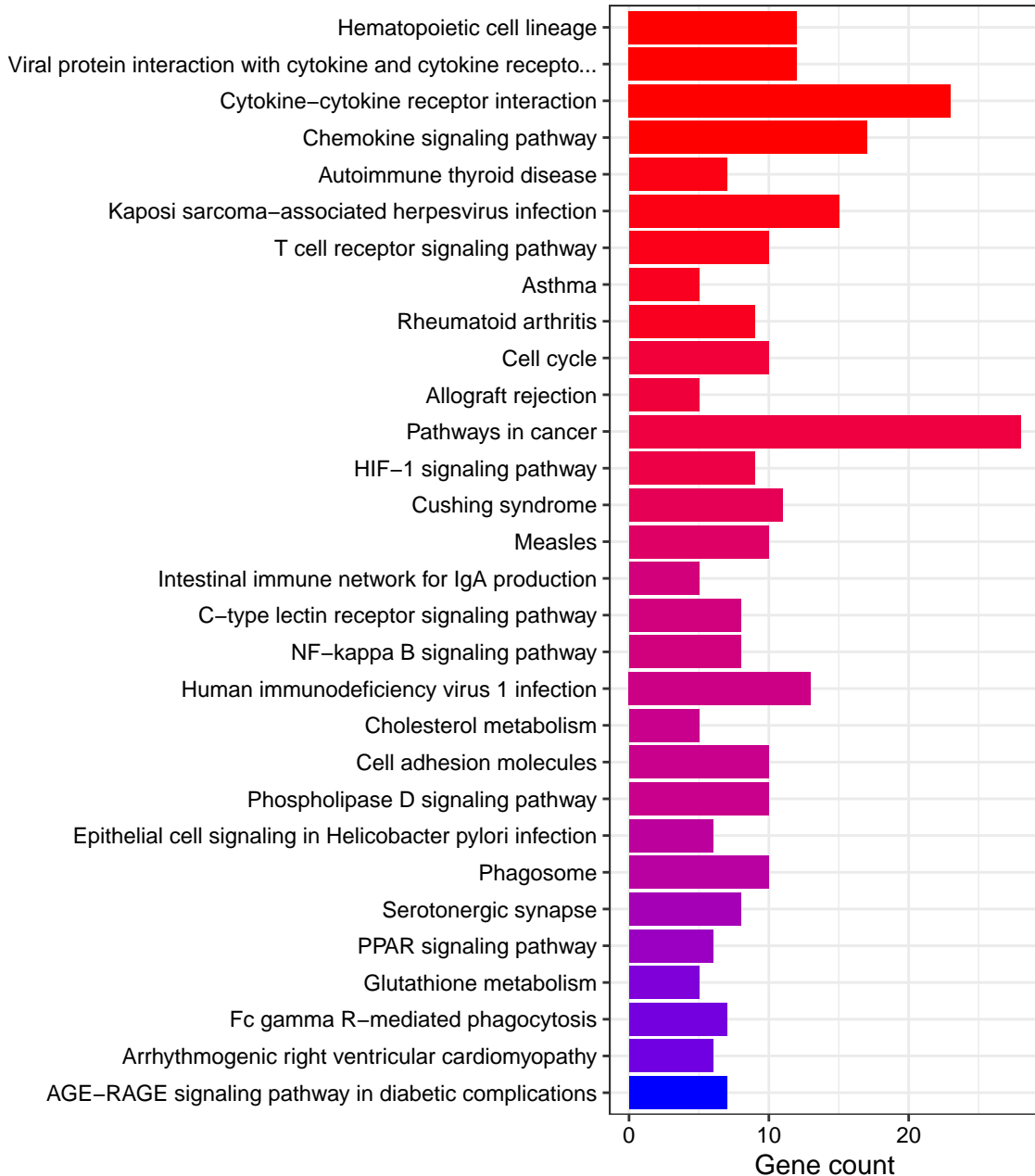

P value

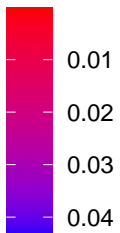

Supplement: Supplementary file 2 [file Data_Sheet_2.ZIP › supplementary2/supplementary2.1_enrich_clusterC/Barplot_ReDisX_GSE59867_clus3_KEGG.pdf]

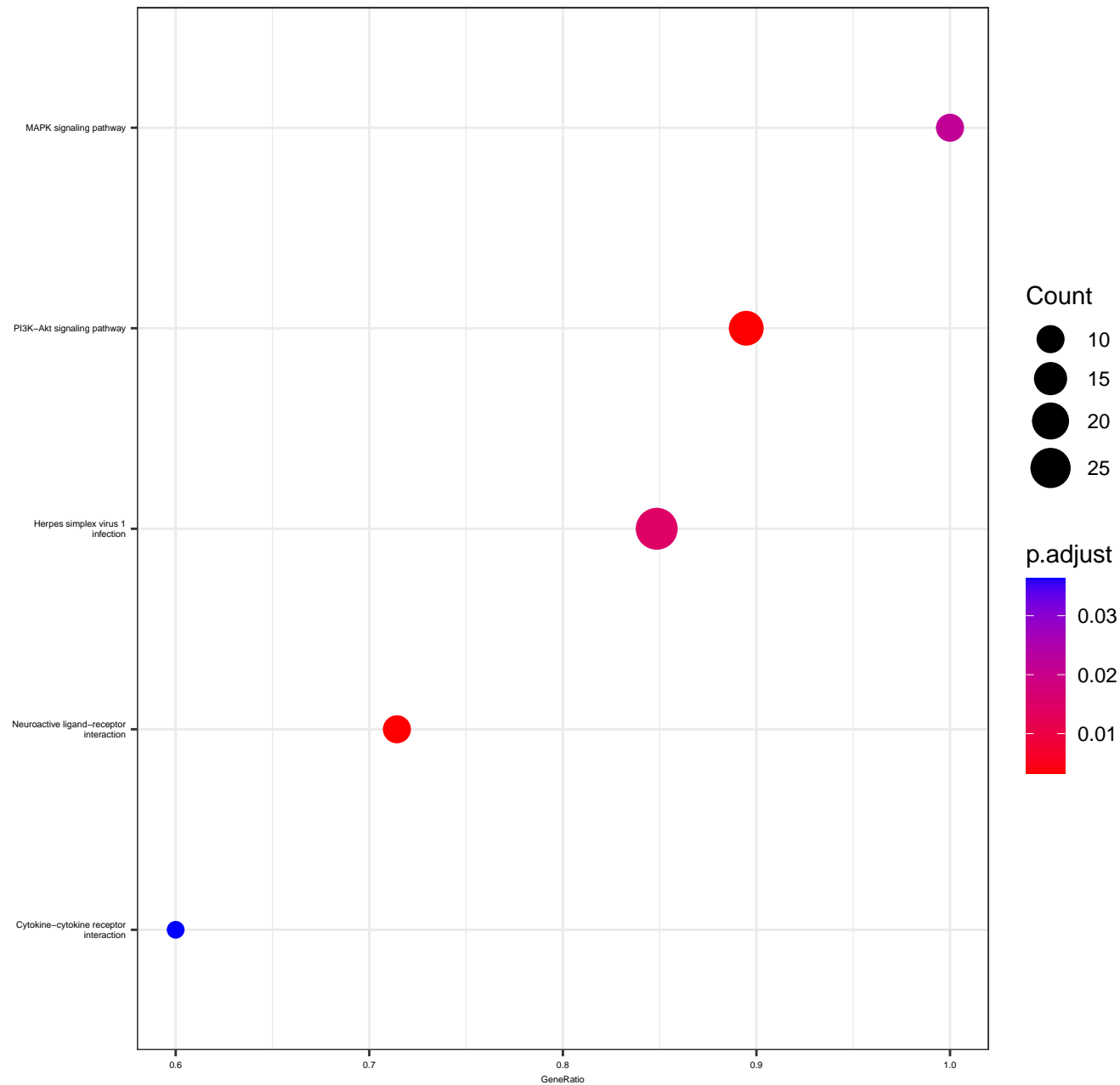

Supplement: Supplementary file 2 [file Data_Sheet_2.ZIP › supplementary2/supplementary2.1_enrich_clusterC/Disgenet_GSE59867_ReDisXclus3_Dotplot.pdf]

# Enrichment analysis by Enrichr

Enriched terms

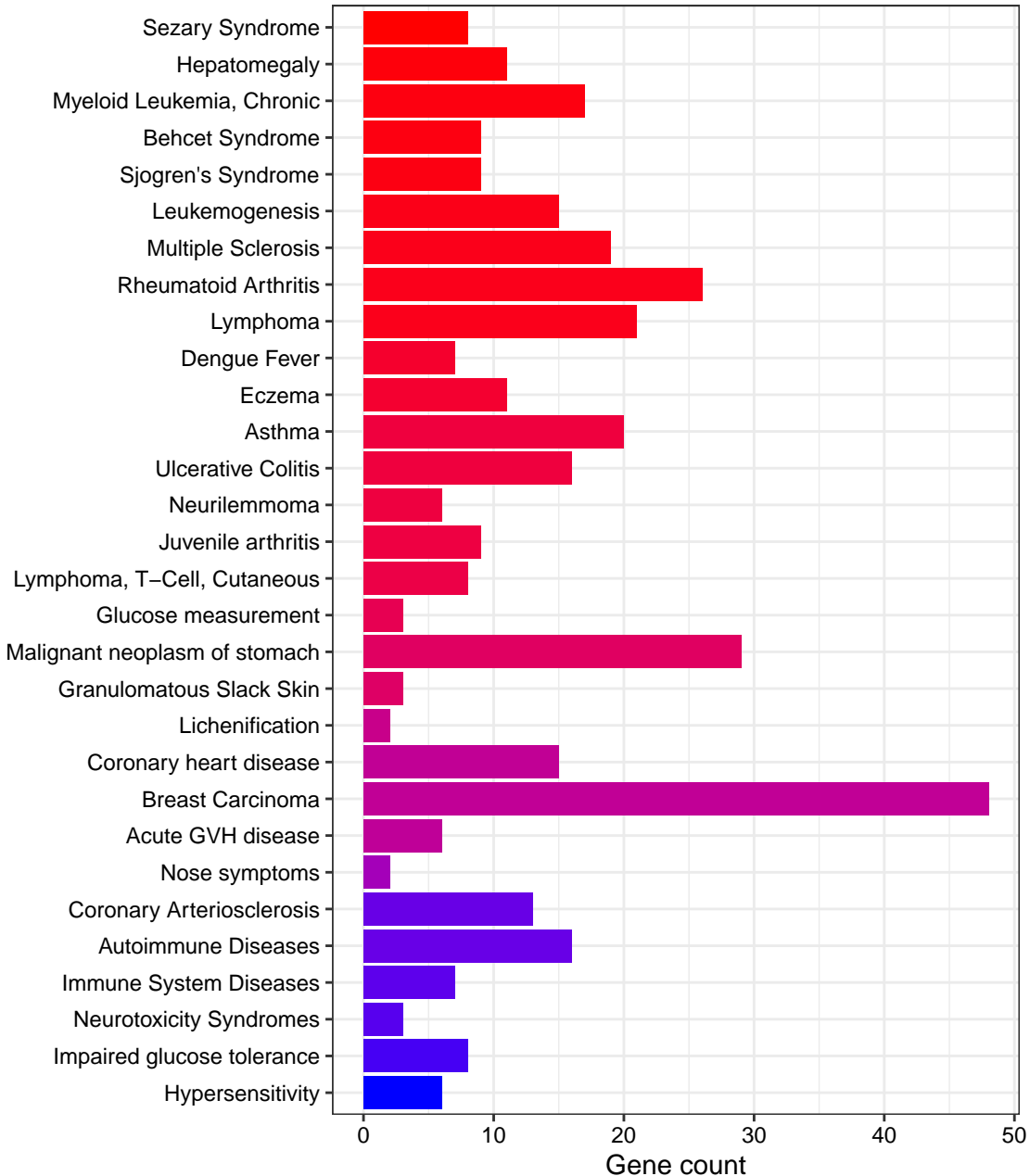

P value

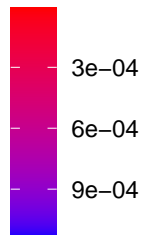

Supplement: Supplementary file 2 [file Data_Sheet_2.ZIP › supplementary2/supplementary2.2_enrich_GSE59867_GSE15573/Barplot_15573_59867_DisGeNet.pdf]

# Enrichment analysis by Enrichr

Enriched terms

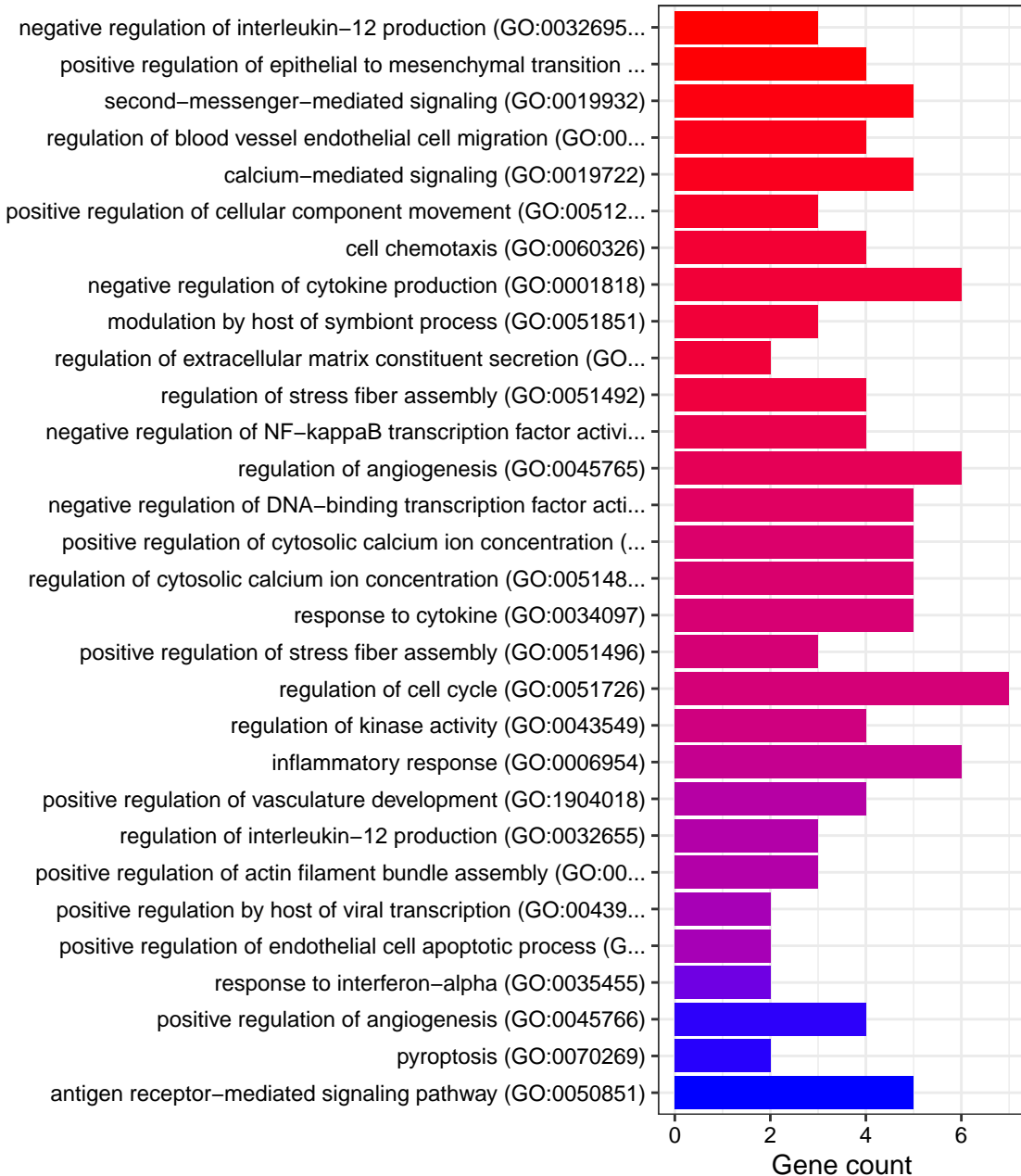

P value

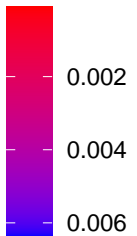

Supplement: Supplementary file 2 [file Data_Sheet_2.ZIP › supplementary2/supplementary2.2_enrich_GSE59867_GSE15573/Barplot_15573_59867_GO_BP.pdf]

# Enrichment analysis by Enrichr

Enriched terms

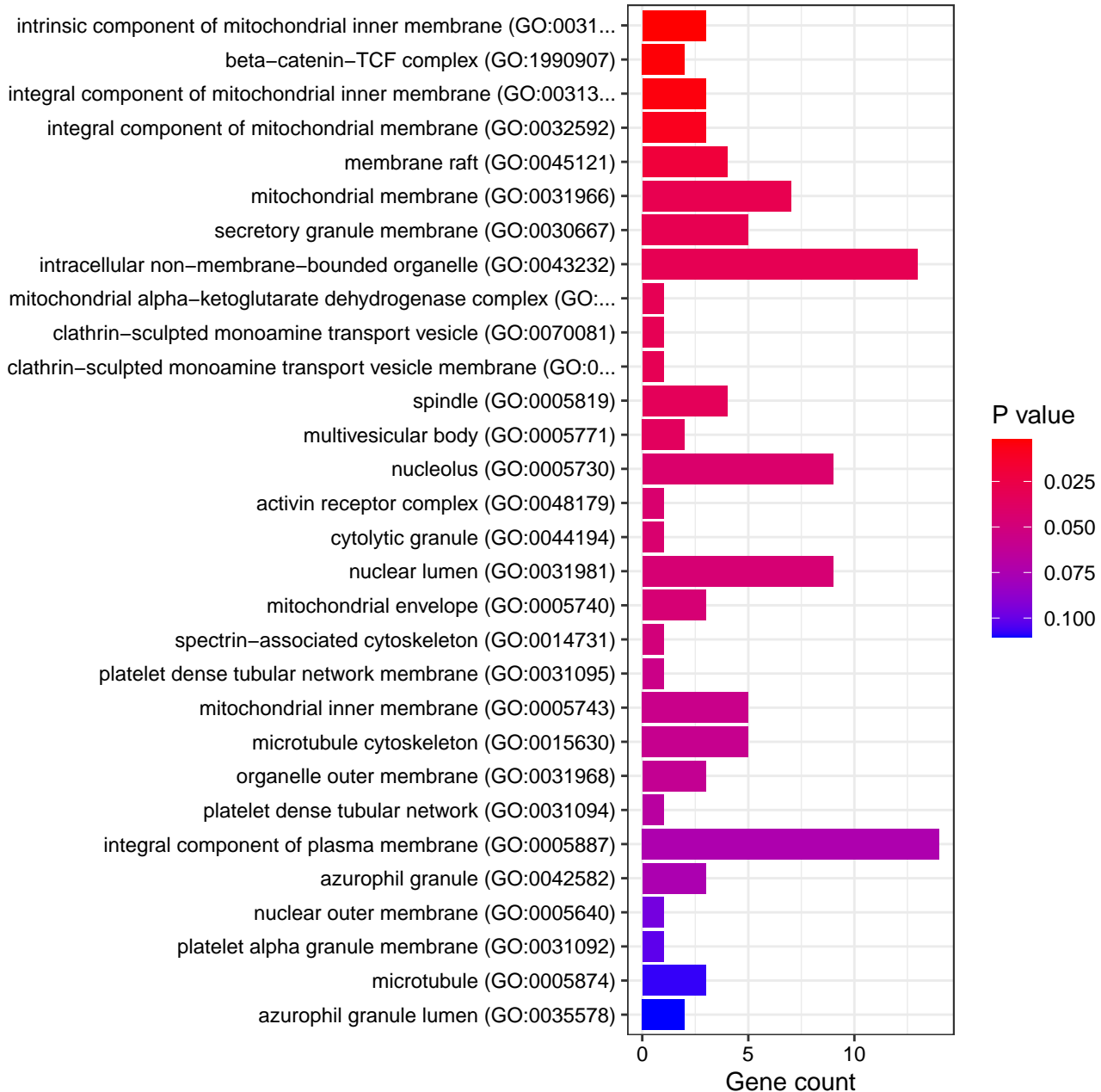

Supplement: Supplementary file 2 [file Data_Sheet_2.ZIP › supplementary2/supplementary2.2_enrich_GSE59867_GSE15573/Barplot_15573_59867_GO_CC.pdf]

# Enrichment analysis by Enrichr

Enriched terms

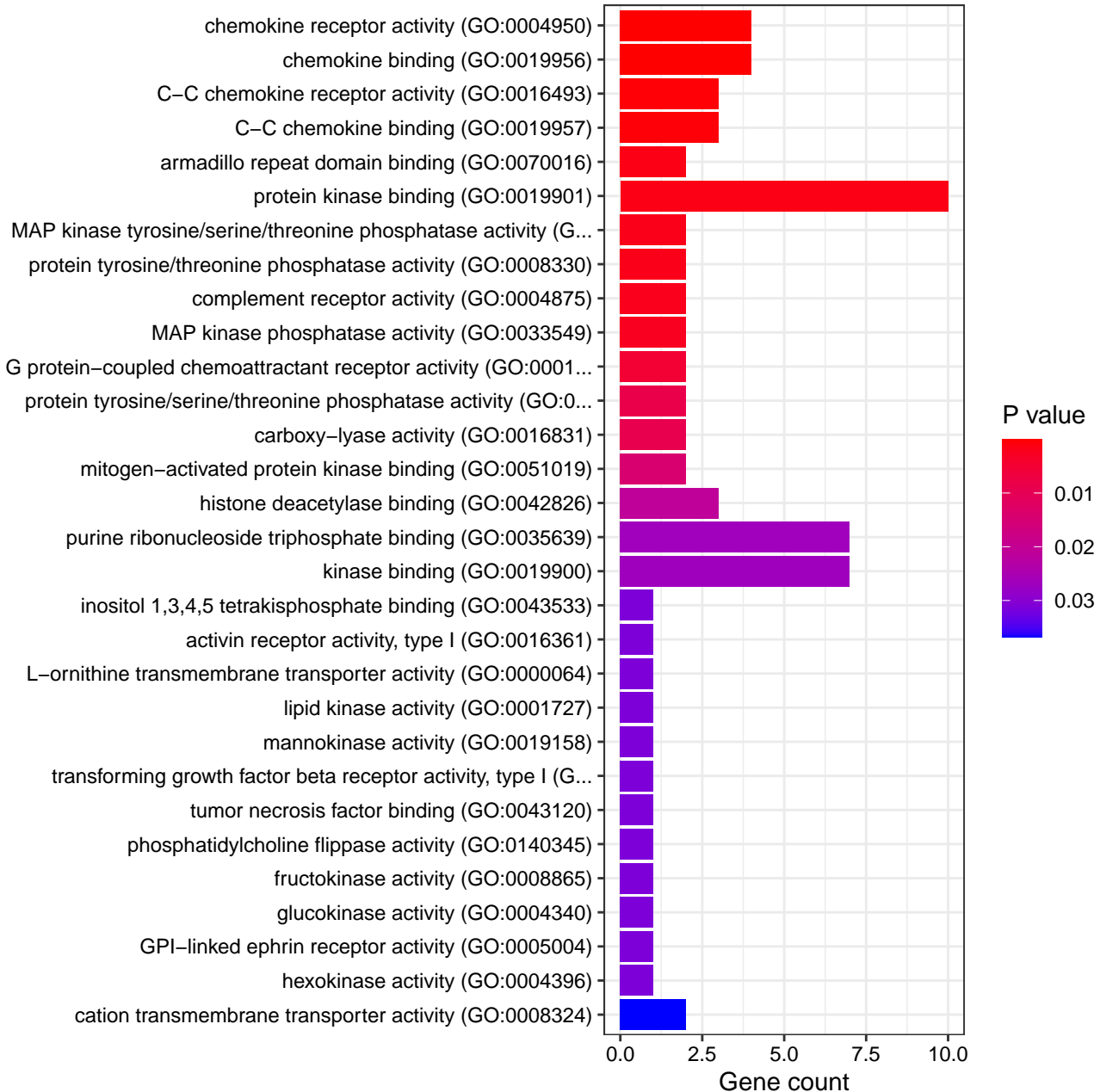

Supplement: Supplementary file 2 [file Data_Sheet_2.ZIP › supplementary2/supplementary2.2_enrich_GSE59867_GSE15573/Barplot_15573_59867_GO_MF.pdf]

# Enrichment analysis by Enrichr

Enriched terms

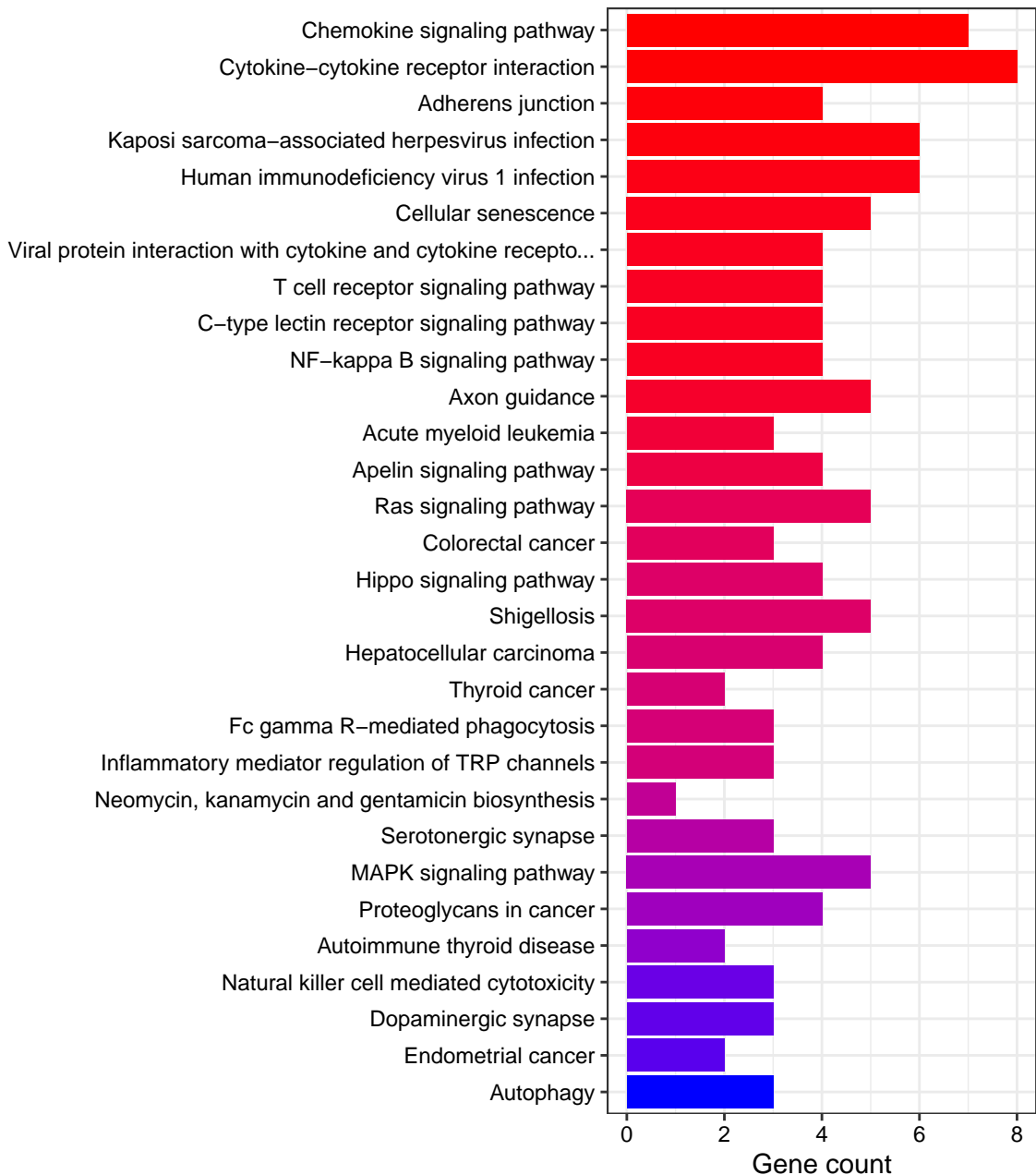

P value

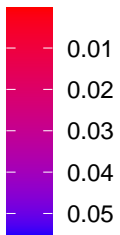

Supplement: Supplementary file 2 [file Data_Sheet_2.ZIP › supplementary2/supplementary2.2_enrich_GSE59867_GSE15573/Barplot_15573_59867_KEGG.pdf]

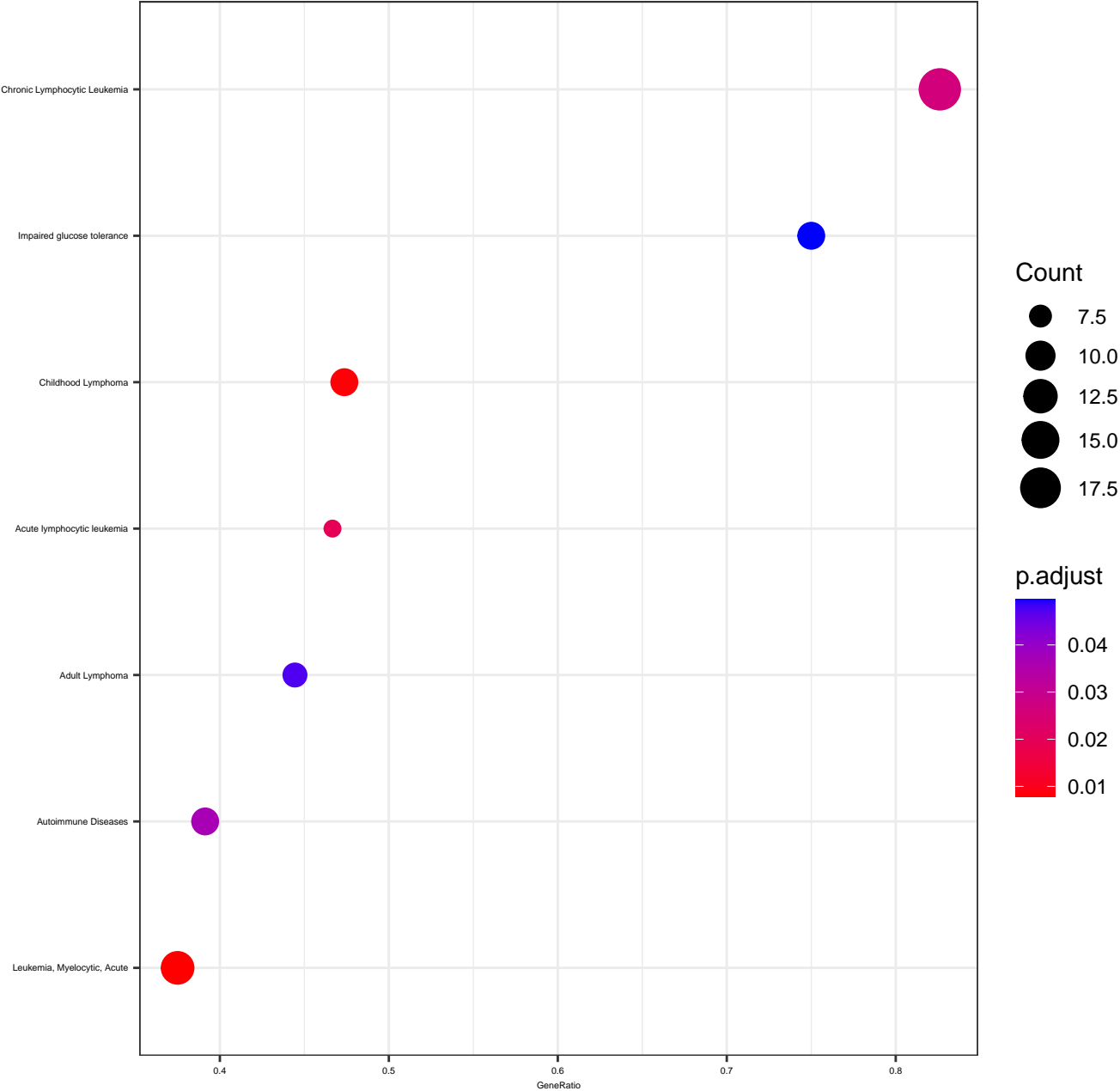

Supplement: Supplementary file 2 [file Data_Sheet_2.ZIP › supplementary2/supplementary2.2_enrich_GSE59867_GSE15573/clusterProfiler_gseDGN_Disgenet_GSE59867_GSE15573_ReDisXclus3_Dotplot.pdf]

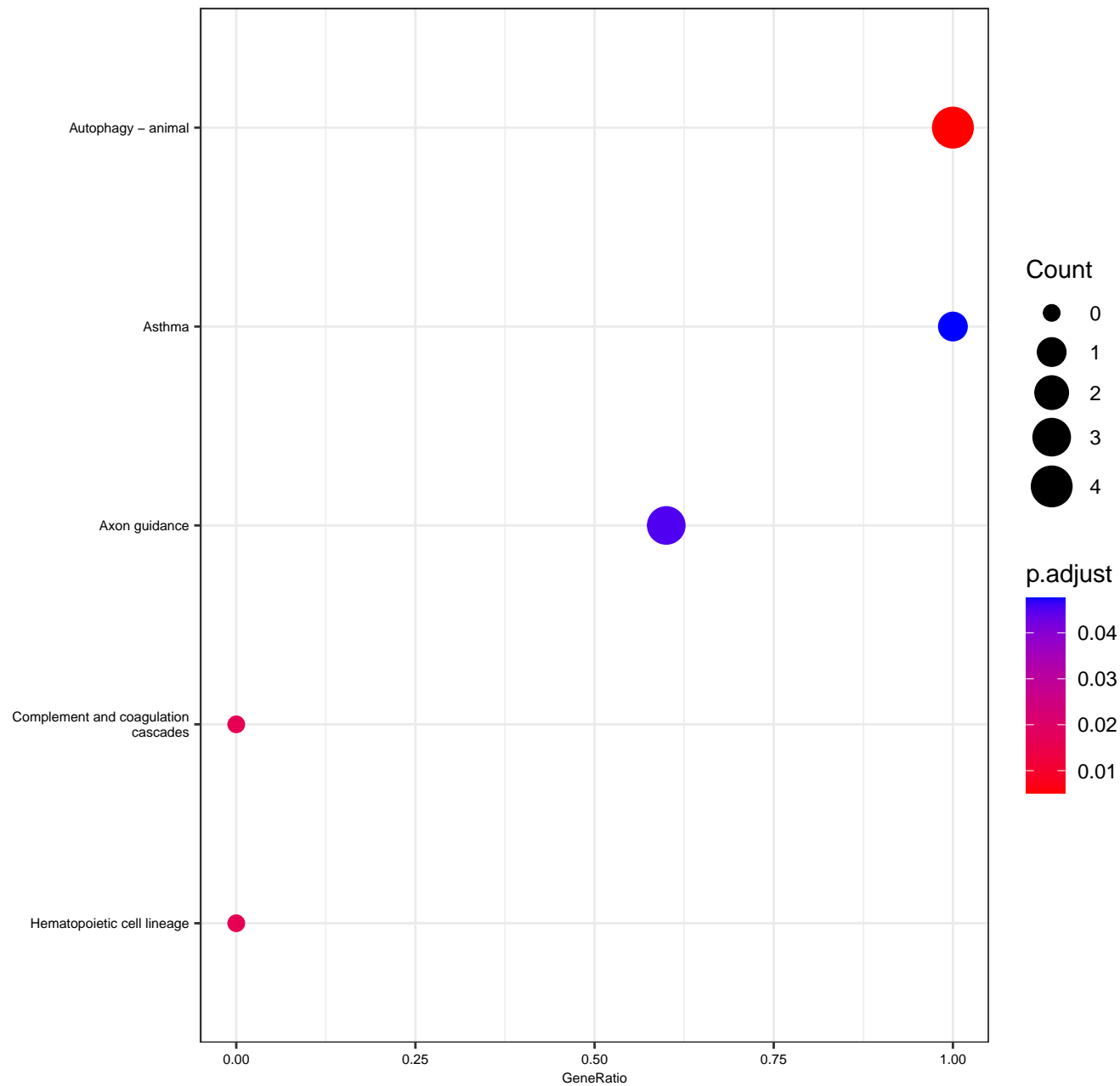

Supplement: Supplementary file 2 [file Data_Sheet_2.ZIP › supplementary2/supplementary2.2_enrich_GSE59867_GSE15573/clusterProfiler_gseKEGG_15573_59867_Dotplot.pdf]

# Enrichment analysis by Enrichr

Enriched terms

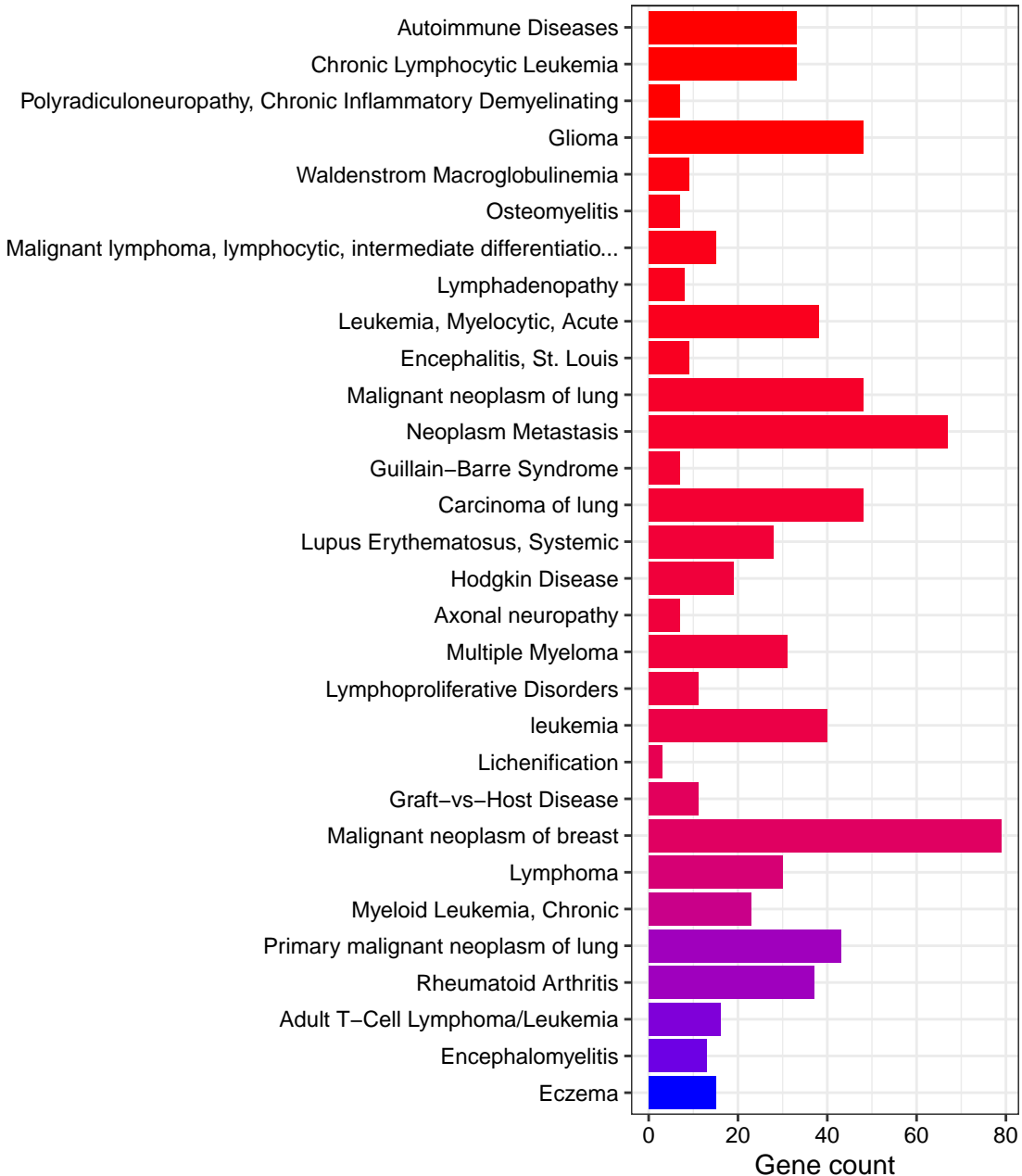

P value

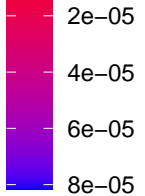

Supplement: Supplementary file 3 [file Data_Sheet_3.ZIP › supplementary3/GSE77298_GSE93272_GSE59867_clus3/enrich_GSE59867_GSE77298/Barplot_77298_59867_DisGeNet.pdf]

# Enrichment analysis by Enrichr

Enriched terms

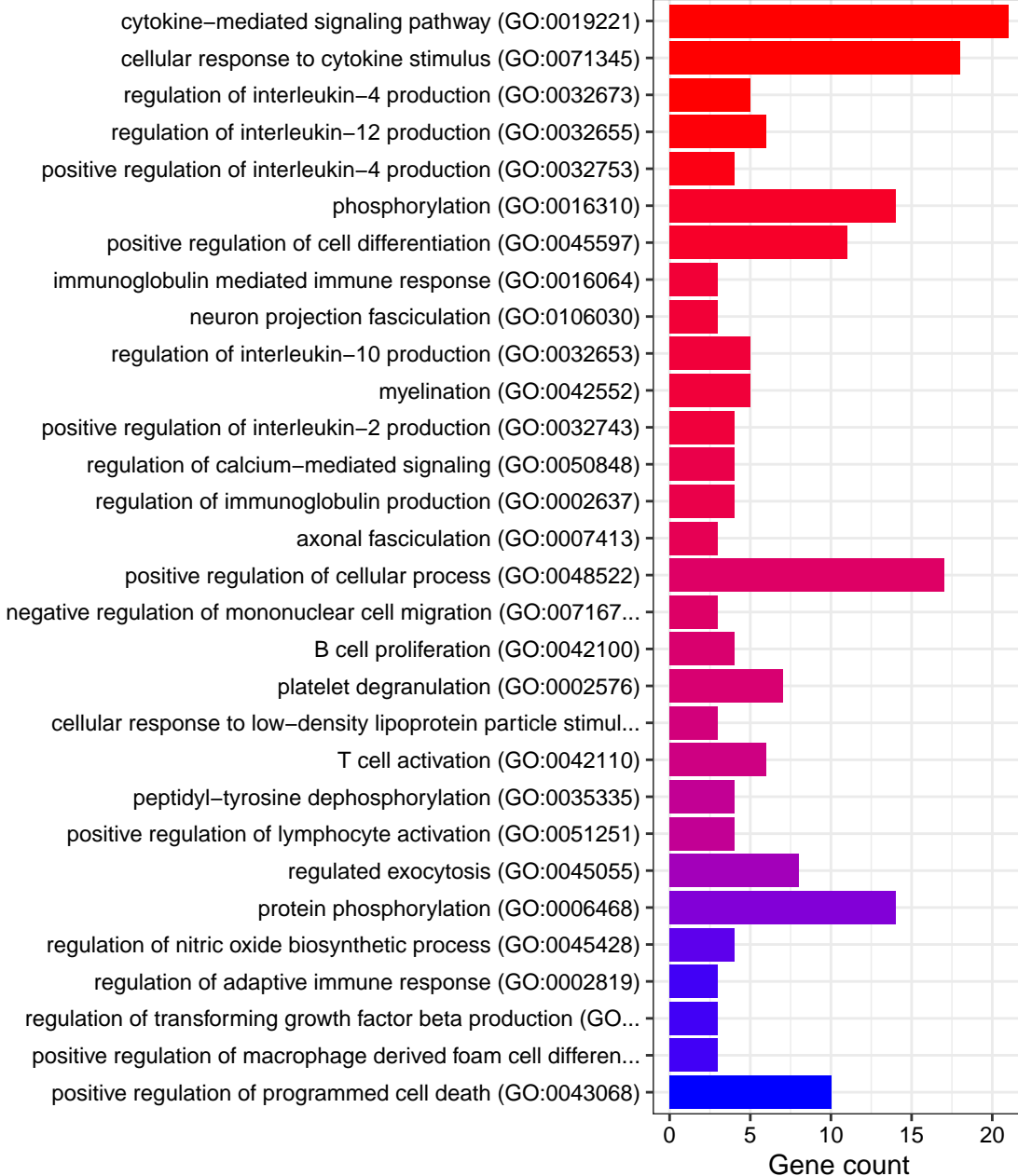

P value

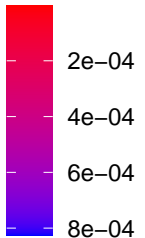

Supplement: Supplementary file 3 [file Data_Sheet_3.ZIP › supplementary3/GSE77298_GSE93272_GSE59867_clus3/enrich_GSE59867_GSE77298/Barplot_77298_59867_GO_BP.pdf]

# Enrichment analysis by Enrichr

Enriched terms

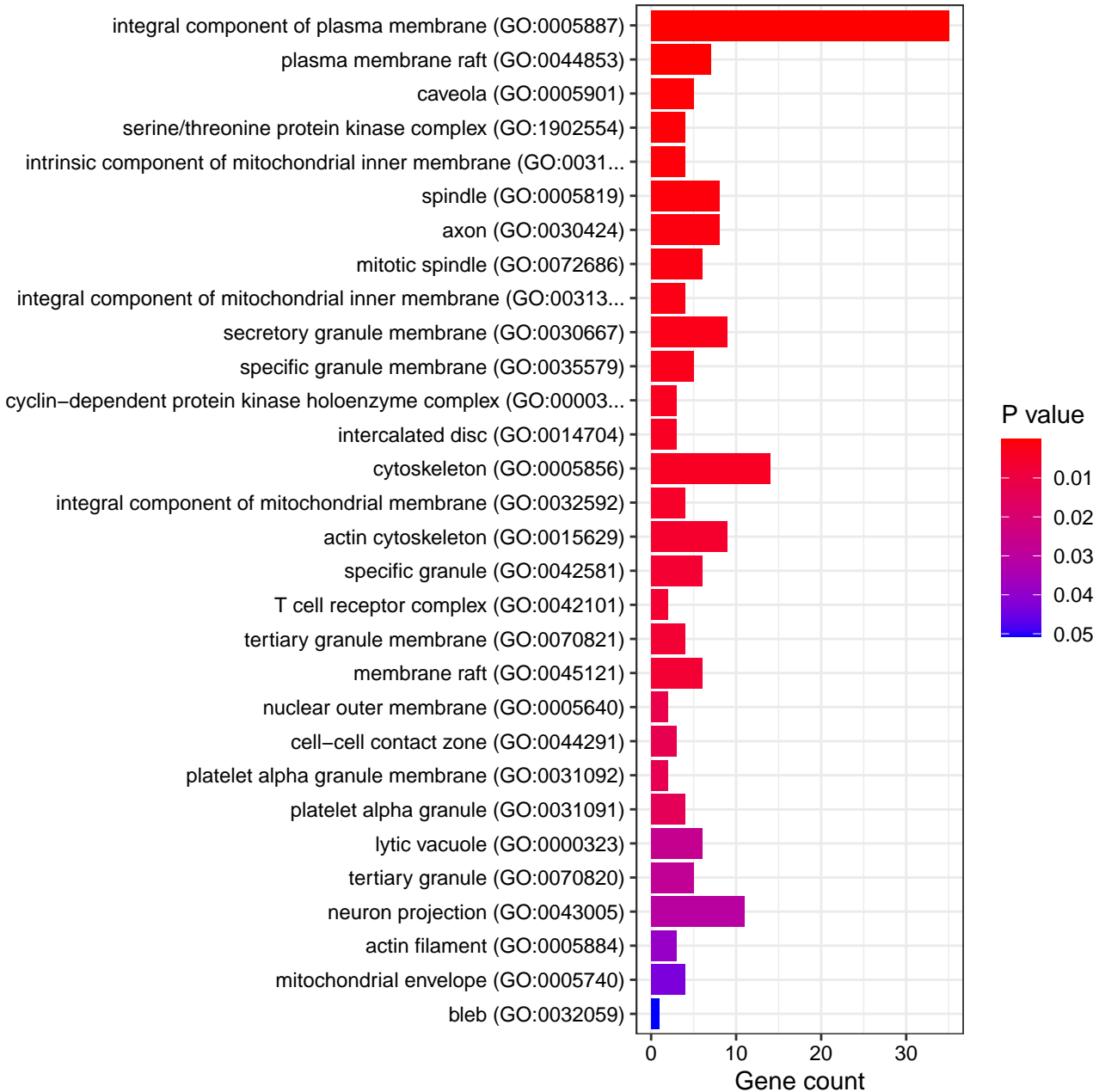

Supplement: Supplementary file 3 [file Data_Sheet_3.ZIP › supplementary3/GSE77298_GSE93272_GSE59867_clus3/enrich_GSE59867_GSE77298/Barplot_77298_59867_GO_CC.pdf]

# Enrichment analysis by Enrichr

Enriched terms

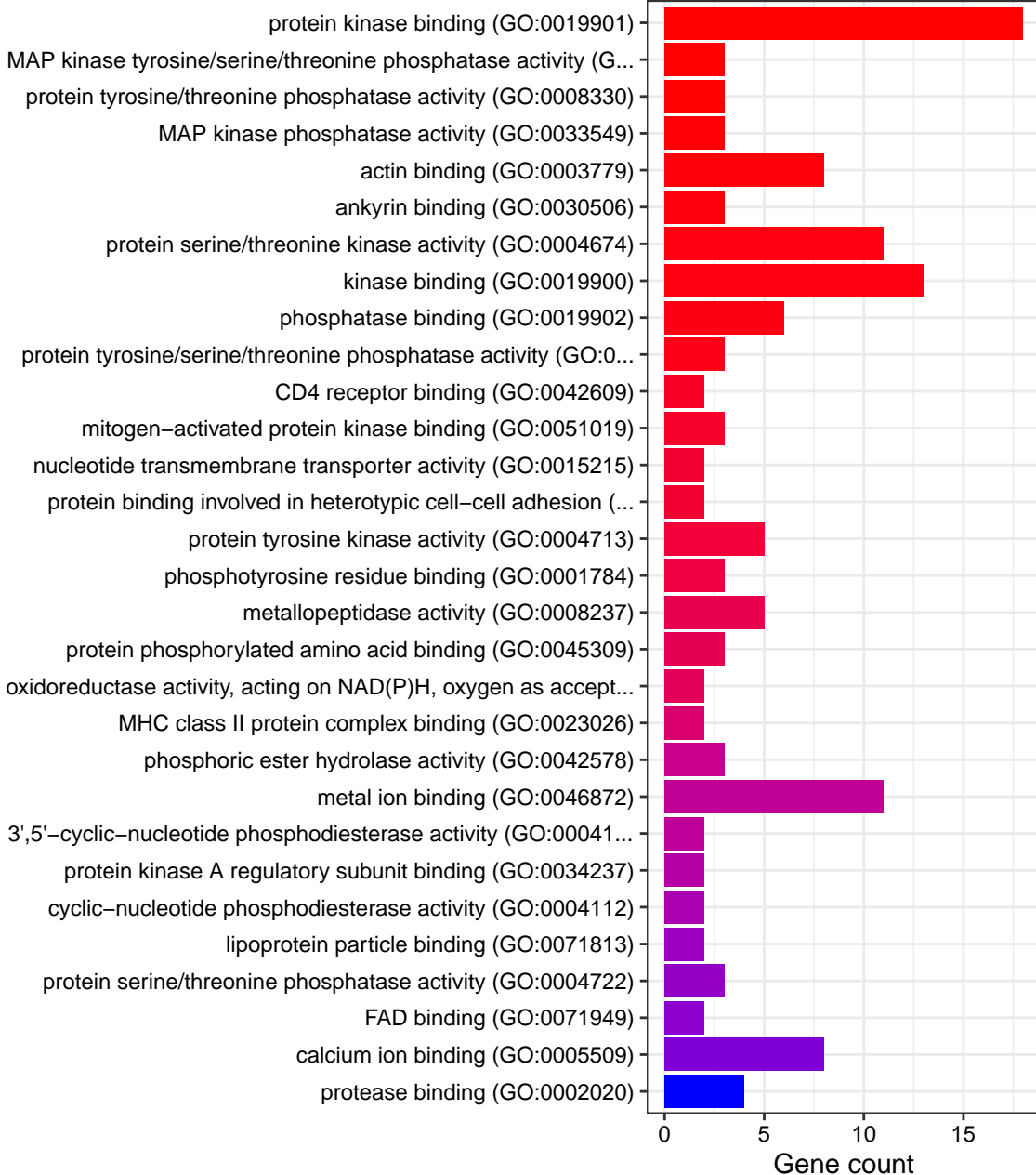

P value

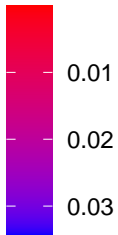

Gene count

Supplement: Supplementary file 3 [file Data_Sheet_3.ZIP › supplementary3/GSE77298_GSE93272_GSE59867_clus3/enrich_GSE59867_GSE77298/Barplot_77298_59867_GO_MF.pdf]

# Enrichment analysis by Enrichr

Enriched terms

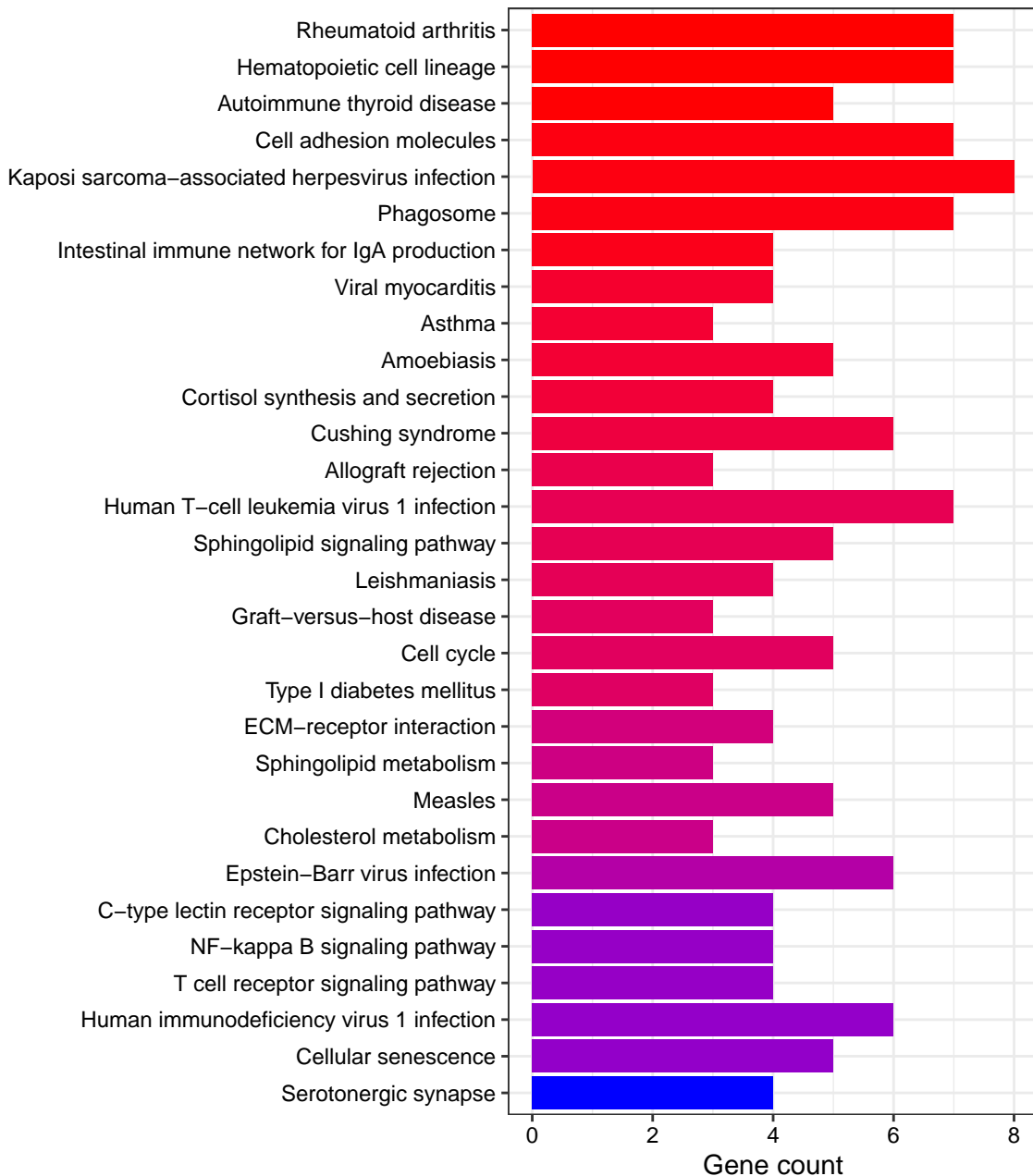

P value

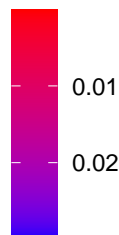

Supplement: Supplementary file 3 [file Data_Sheet_3.ZIP › supplementary3/GSE77298_GSE93272_GSE59867_clus3/enrich_GSE59867_GSE77298/Barplot_77298_59867_KEGG.pdf]
